# Fragment-based drug design of a bacterial kinase inhibitor capable of increasing the antibiotic sensitivity of clinical isolates

**DOI:** 10.1038/s42004-025-01795-6

**Published:** 2025-11-27

**Authors:** Julien Kowalewski, Robin Deutscher, Marion Richardoz, Mathilde Tomaszczyk, Muriel Gelin, Gilles Labesse, Felix Hausch, Gerard D. Wright, Catherine Dunyach-Remy, Jean-François Guichou, Corinne Lionne

**Affiliations:** 1 Centre de Biologie Structurale (CBS), University of Montpellier, CNRS UMR 5048, INSERM U1054, Montpellier, France; 2 Institute for Organic Chemistry and Biochemistry, Technical University of Darmstadt, Darmstadt, Germany; 3 Virulence Bactérienne et Infections Chroniques, INSERM U1047, Department of Microbiology and Hospital Hygiene, University of Montpellier, Nîmes, France; 4 Centre for Synthetic Biology, Technical University of Darmstadt, Darmstadt, Germany; 5 M.G. DeGroote Institute for Infectious Disease Research, Department of Biochemistry and Biomedical Sciences, McMaster University, Hamilton, ON Canada

**Keywords:** Drug screening, X-ray crystallography, Transferases, Screening, Kinases

## Abstract

According to the World Health Organization (WHO), antimicrobial resistance is a serious global health issue. Overcoming antibiotic resistance involves several strategies, including the inhibition of resistance mechanisms. Among the various resistance mechanisms, aminoglycoside phosphotransferases (APHs) catalyze the transfer of the γ-phosphate from a nucleotide donor to various aminoglycosides, leading to their inactivation. In this work, using a fragment-based drug design (FBDD) approach, we have identified and characterized a promising APH inhibitor capable of increasing the sensitivity of *Pseudomonas aeruginosa* and *Staphylococcus aureus* resistant to aminoglycosides. It is therefore a good candidate for the future development of APH inhibitors to be prescribed in combination with aminoglycosides. This molecule is a competitive inhibitor of adenosine 5’-triphosphate (ATP), the phosphate donor of APHs. Further studies are required to optimize this molecule to improve its specificity for APHs and its bioavailability in bacteria.

## Abstract (web summary)

Antimicrobial resistance poses a significant global health challenge, with aminoglycoside phosphotransferases (APHs) contributing to antibiotic inactivation. Here, the authors employ fragment-based drug design to identify a promising APH inhibitor, enhancing bacterial sensitivity to aminoglycosides.

## Introduction

The discovery of the first antibiotic in the 1930s revolutionized human health, making it possible to treat bacterial infections, which at the time represented one of the world’s leading causes of death^1^. Used extensively during World War II, penicillin contributed significantly to the Allied victory and saved the lives of many soldiers. Bacterial resistance to antibiotics has evolved in response to the constant selective pressure exerted by antimicrobial agents, and the overuse of antibiotics has exacerbated this issue^2^. By 2050, antibiotic multidrug resistance could be responsible for ten million deaths a year^3^. One success in countering penicillin resistance is the concomitant use of β-lactam and an inhibitor of the bacterial enzymes that degrade the antibiotic, β-lactamases^4^.

Aminoglycosides are another class of antibiotics which are clinically used to treat a wide variety of bacterial infections^5–7^. For example, Gentamicin and Tobramycin are used in the treatment of pneumonia and meningitis caused by Gram-negative bacilli and nosocomial infections by Gram-positive bacteria; Amikacin is used in the treatment of serious nosocomial Gram-negative bacterial infections and mycobacterial infections in acquired immunodeficiency syndrome (AIDS) patients. Like all classes of antibiotics, aminoglycosides are subject to bacterial resistance. General mechanisms of antibiotic resistance can be divided into four groups: (1) poor membrane permeability, which prevents the antibiotic from getting into the bacteria and reaching its target; (2) active efflux pumps that reject the antibiotic from the bacteria; (3) modification of the antibiotic target (mutation or methylation); (4) enzymes inactivating the antibiotics^8^.

APHs are such enzymes that catalyze the transfer of the γ-phosphate from a nucleotide triphosphate (NTP) to an aminoglycoside molecule^9^. The phosphorylation of aminoglycosides leads to the addition of a negatively charged group, which interferes with the interaction of the antibiotics with their main target, the ribosome. Despite their low sequence identity, this family of enzymes share a high structural similarity^10^. These globular enzymes can be divided into three different domains: an amino-terminal domain where the NTP binding site is located, a core domain linking the amino-terminal domain to the carboxy-terminal domain and the carboxy-terminal domain where the aminoglycoside binds.

The N-terminal domain is characterized by a five-stranded β-sheet, which is conserved in many kinases and covers the NTP-binding cavity. The binding and choice of NTP is governed by several amino acids present in the cavity, and even if some of them are not strictly conserved, their properties are preserved. An extended linker constitutes a hinge and interacts with the nucleobase of the NTP. Backbone atoms of one or two residues on this hinge form hydrogen bonds with the nucleobase. Consequently, the side chains of these residues are variable. Hydrophobic residues located in the β-sheet and a loop not very conserved allow the nucleobase to be stabilized in the cavity by van der Waals interactions and/or π-stacking. In addition, a residue known as the gatekeeper is located deep in the binding site, and its nature allows the fixation of ATP, guanosine 5’-triphosphate (GTP), or both to the protein. Finally, three catalytic residues are involved in the transfer of the γ-phosphate from the NTP to the aminoglycoside molecule. The catalytic triad is highly conserved and consists of one positively charged residue and two negatively charged residues: a lysine, a glutamic acid and an aspartic acid. In some cases, the lysine is replaced by an arginine, as in the case of APH(2”)-IIIa from *Enterococcus gallinarum*. The nucleotide binding site is the most structurally conserved domain between APHs, but also atypical protein kinases^11^. As a consequence, protein kinase inhibitors can inhibit APHs and restore antibiotic sensitivity^12–14^. This motivated our search for ATP-competitive and APH-specific inhibitors to restore bacterial susceptibility to aminoglycosides, following the example of β-lactamase inhibitors.

For our primary FBDD screening, we chose APH(2”)-IVa from *Enterococcus casseliflavus* since it confers a high level of resistance in *Enterococcus* towards various aminoglycosides that are prescribed in clinics^15^. This APH has the particularity of being able to use ATP or GTP as a phosphate donor^16,17^. After a structure-activity relationship on catalogue, we finally identified a hit compound which can inhibit in vitro several clinically relevant APHs and increase the sensitivity of multidrug-resistant clinical isolates to aminoglycosides.

## Results

### Primary fragment screening

We studied the impact of 389 fragments on the thermal stability and in vitro enzymatic activity of APH(2”)-IVa. These fragments come from our own chemical library, which consists of compounds containing nitrogen heterocycles most frequently found in approved drugs^18,19^. They were chosen to meet most of the classical physicochemical criteria of the fragments^20^ and to offer a wide chemical diversity, thus covering a large chemical space^21^.

To perform this primary screening, we tested these molecules at a relatively high concentration of 500 µM, which is standard for screening very small molecules such as fragments^22^. Results are presented in Supplementary Fig. 1. Not surprisingly, we were not able to demonstrate a strict correlation between the impact of the fragments tested on the melting temperature, Δ*T*_m_, measured by Thermal Shift Assay (TSA), and the percentage of enzymatic activity inhibition. Nevertheless, it seems that when a fragment induces a positive Δ*T*_m_ by more than one degree, it is more likely to induce significant inhibition of the enzyme. From the primary screening of these 389 fragments by TSA, we retained 9 fragments: 5 fragments inducing a Δ*T*_m_ > 1 °C and 4 fragments inducing a Δ*T*_m_ < −1 °C. In the screening of the same fragments by activity assay, we retained 39 fragments inhibiting APH(2”)-IVa by at least 25%, 5 of which were in common with selection by TSA. As a result, a total of 43 fragments (Table 1) were selected from the 389 tested, representing a hit rate of approximately 12%, which is typical for the FBDD approach^23^. The most common motif among the selected hits is azaindole, found in 7 fragments: F382, F381, F352, F340, F385, F337, and F354 (highlighted with a purple background in Table 1).

**Table 1:**
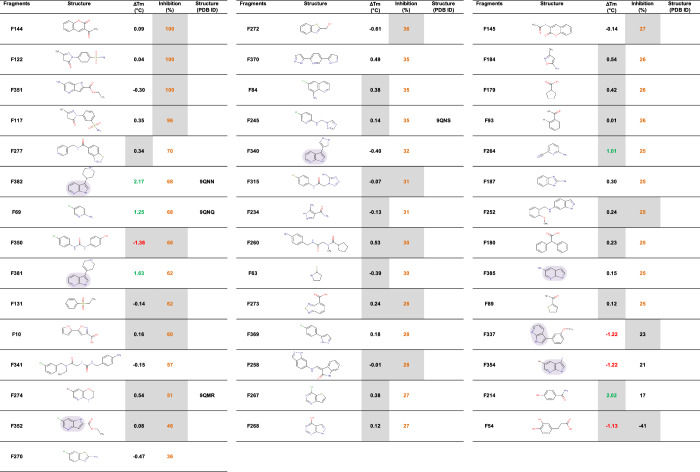
Fragments selected after the primary screening of 389 fragments at 500 µM on APH(2”)-IVa by TSA and activity assays

Δ*T*_m_ > 1 °C are indicated in green, Δ*T*_m_ < −1 °C in red and inhibition ≥ 25% in orange. Selected fragments are ranked from highest to lowest inhibition. The gray boxes indicate that the compound absorbs or fluoresces at the measurement wavelengths, which may distort the result. The purple backgrounds highlight the azaindole motifs.

### Identification of scaffolds interacting with the hinge

We carried out X-ray crystallography screening of these 43 most promising binders. Determining the structures enabled us to identify their binding sites and obtain precise information on their interactions with protein residues.

**Fig. 1:**
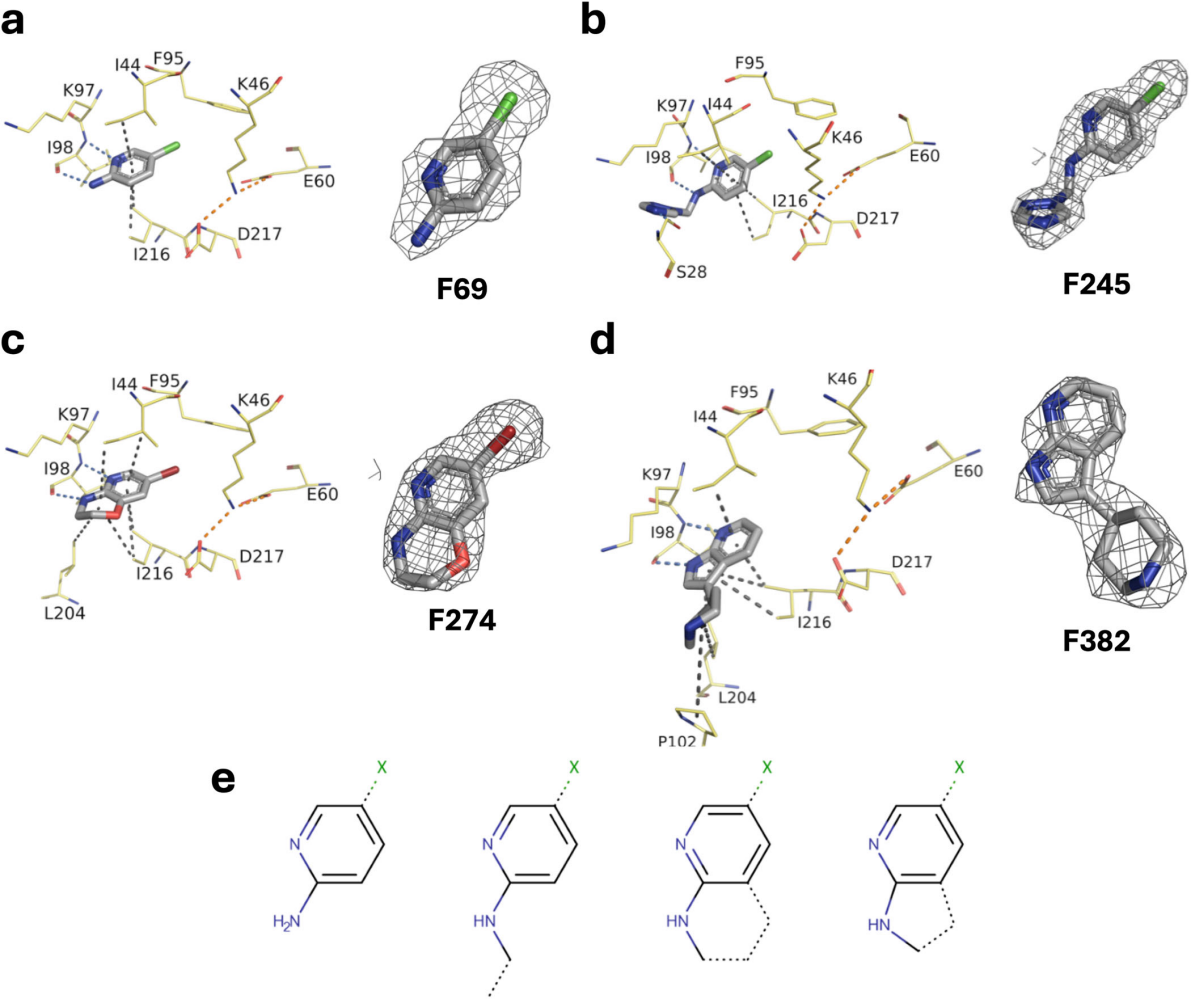
Crystal structures of APH(2”)-IVa in complex with 4 fragments selected during primary screen. Crystal structures and corresponding omit maps contoured at a sigma level ± 1 of APH(2”)-IVa in complex with (**a**) **F69** determined at 2.38 Å (PDB 9QNQ), (**b**) **F245** at 2.00 Å (PDB 9QNS), (**c**) **F274** at 1.78 Å (PDB 9QMR) and (**d**) **F382** at 2.65 Å (PDB 9QNN). Inhibitors are represented in gray sticks and residues involved in interactions are shown as yellow lines. Interactions are shown as dashed lines: van der Waals interactions in gray, hydrogen bonds in blue and ionic bonds in orange. **e** Minimal scaffolds identified for optimal interaction with the hinge of APH(2”)-IVa, where X represents a halogen atom.

The first structures obtained indicate that four fragments interact at the nucleotide interaction site, specifically at the hinge. The ionic interactions between the three catalytic residues, Lys46, Glu60, and Asp217, are preserved in all four complexes (orange dashed lines in Fig. 1a*–*d). There are three types of common interactions involved between the protein and these four fragments. First, Ile44 and Ile216 stack the aromatic heterocycles of the fragments (black dashed lines). Second, the nitrogen of the backbone of Ile98 forms a hydrogen bond with the nitrogen of the pyridine motif found in all four fragments (blue dashed lines). Finally, the carbonyl of Ile98 forms a hydrogen bond with the primary amine of the pyridine of **F69** and **F245** or the amine in the oxazine of **F274** or in the pyrrole of **F382**. Three of these fragments have a halogen atom that points to the gatekeeper residue Phe95. The fact that **F382** strongly stabilizes and inhibits APH, but does not have a halogen, raises questions about the importance of this atom for the interaction. Minimal scaffolds were identified for optimal interaction with the hinge residues of APH(2”)-IVa (Fig. 1e).

We then searched our library of fragments for molecules that possessed these minimum motifs and attempted to crystallize them in complexes with APH. This led to two new structures of APH in complex with fragments **F136** and **F355**, both of which contain an azaindole motif (Supplementary Fig. 2).

### The presence of a halogen atom on the minimal scaffolds greatly increases the inhibition

In order to verify whether the presence of a halogen atom in position 5 of the pyridine or of the 7-azaindole is important for APH inhibition, we ordered a series of halogen-containing analogues of these two scaffolds. In the rest of the text, the fragments initially present in our initial library retain their numbering from **F1** to **F389**, whereas analogue molecules purchased subsequently are named by just a number.

**Fig. 2:**
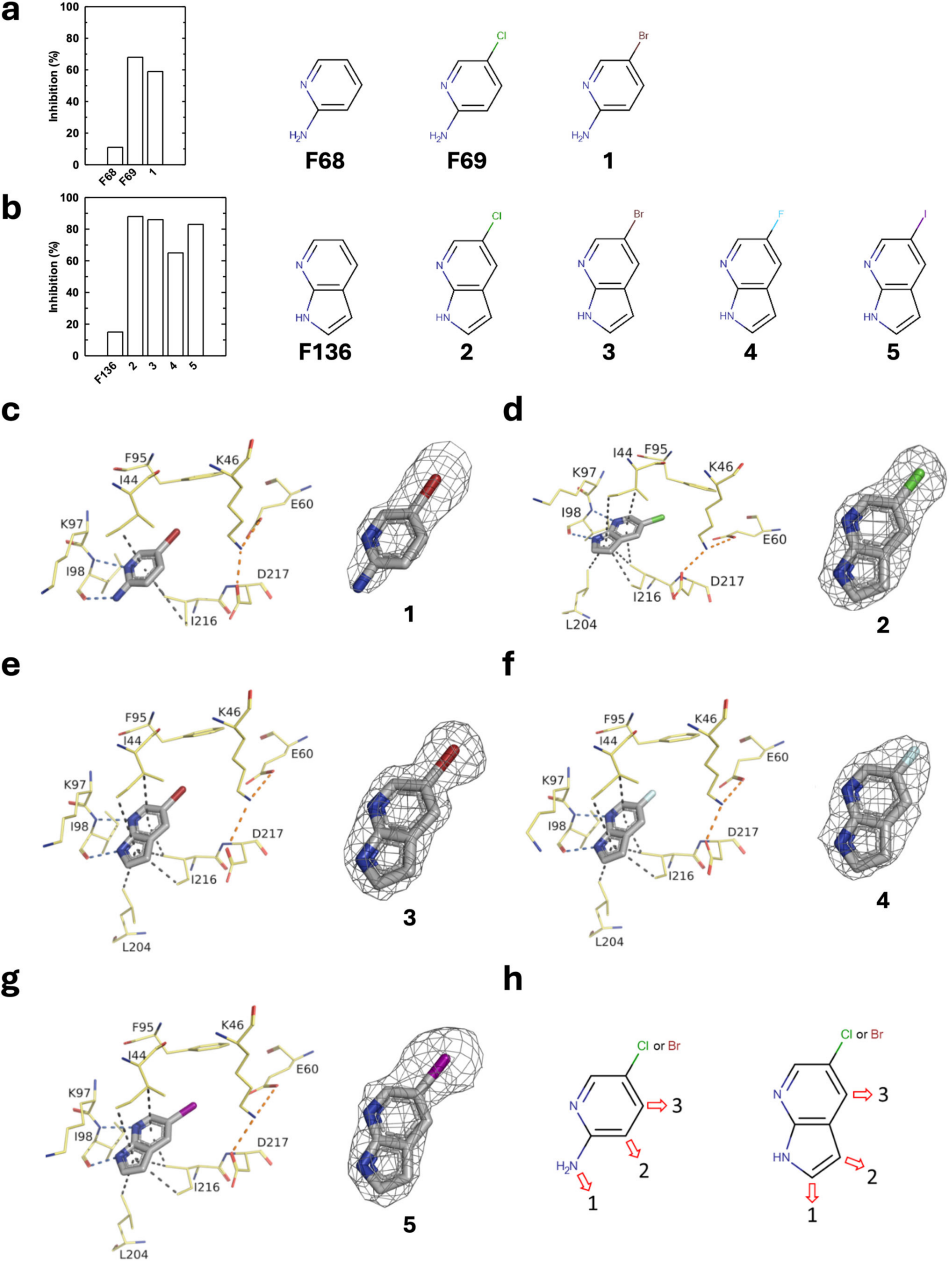
Characterization of halogen-containing analogues of identified scaffolds interacting with the hinge of APH and potential vectors for hit optimization. **a**, **b** APH(2”)-IVa enzymatic activity inhibition by 500 µM of fragments and structure of the analogues tested. **c**–**g** Crystal structures and corresponding omit maps contoured at a sigma level of ± 1 of APH(2”)-IVa in complex with **1** at 2.47 Å (PDB 9QNW), **2** at 1.88 Å (PDB 9QOK), **3** at 1.86 Å (PDB 9QPB), **4** at 2.13 Å (PDB 9QOL) and **5** at 2.13 Å (PDB 9QOM). Inhibitors are represented in gray sticks and residues involved in interactions are shown as yellow lines. Interactions are shown as dashed lines: van der Waals interactions in gray, hydrogen bonds in blue and ionic bonds in orange. **h** Potential vectors, represented as red arrows, for optimization of the most promising hits, **F69** and **2**.

We compared the APH inhibition by these different analogues and obtained crystal structures of the corresponding complexes (Fig. 2). The presence of a chlorine atom at position 5 of the minimal fragments, pyridine **F68** or 7-azaindole **F136**, resulted in a significant increase of the inhibition of APH(2”)-IVa. Indeed, at 500 µM, **F69** inhibited enzyme activity by 68%, i.e. 6.2 times more than **F68** (Fig. 2a). The same applied to **2** with 88% inhibition, i.e. 5.9 times more than **F136** (Fig. 2b). Of the various halogens tested, chlorine and bromide appeared to be the most effective in terms of inhibition.

Crystallographic structures of APH(2”)-IVa in complex with these new analogues confirmed that these fragments also interact with the hinge residues in the nucleotide binding site, as well as with the hydrophobic residues that usually stack with the nucleobase moiety (Fig. 2c–g). The presence of a halogen atom in the fragments seems to impose a defined orientation in the binding site, with the halogen atom pointing toward the gatekeeper residue Phe95. Indeed, the non-halogenated 7-azaindole, **F136**, seems to adopt alternating orientations in the active site (Supplementary Fig. 2a, b), and the 3-hydroxymethyl-7-azaindole, **F355**, is in an inverted position compared with other halogenated azaindole compounds (Supplementary Fig. 2c). The binding mode of all halogenated molecules is similar, and interactions with protein residues are conserved, as shown by the crystallographic structures determined for these compounds in complexes with APH(2”)-IVa (Fig. 2c–g). This is true in the two independent polypeptide chains in the crystal asymmetric unit.

Based on these structures, we identified three potential vectors for optimization of 2-amino-5-(chloro/bromo)pyridine or 5-(chloro/bromo)-7-azaindole (Fig. 2h). We therefore searched and ordered commercial analogues of these motifs bearing one or more substitutions at the positions indicated by the arrows.

### Extensions on vector 3 of F69 bring new interactions with APH

We ordered 65 commercially available analogues of 5-(chloro/bromo)pyridine. 31 molecules contained exactly this motif, including 7 compounds with a substitution according to vector 1, 14 compounds according to vector 2, 8 compounds according to vector 3, and 2 compounds modified in two different positions. The other 34 molecules contained a similar, but slightly distinct core, and various substitutions. All were tested at 500 µM by TSA and activity assay. The parent fragments and the selected analogues based on Δ*T*_m_ and inhibition are shown in Table 2.

**Table 2:**
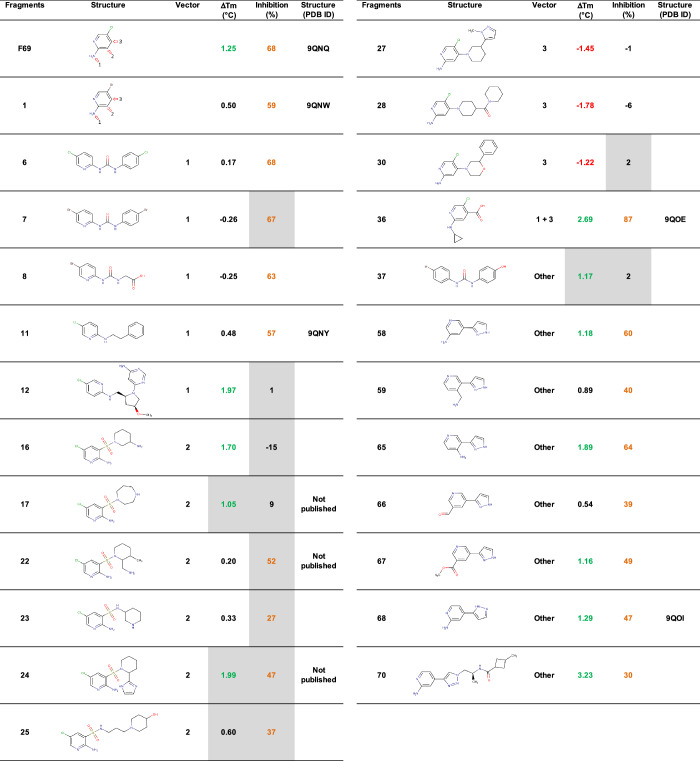
Analogues of 2-amino-5-(chloro/bromo)pyridine selected after assaying at 500 µM on APH(2”)-IVa by TSA and activity assays

Δ*T*_m_ > 1 °C are indicated in green, Δ*T*_m_ < −1 °C in red and inhibition ≥ 25% in orange. The gray boxes indicate that the compound absorbs or fluoresces at the measurement wavelengths, which may distort the result. Analogues are classified by optimization vector and were selected because they modified the *T*_m_ by more than 1 °C and/or inhibited the APH activity by more than 25%.

**Fig. 3:**
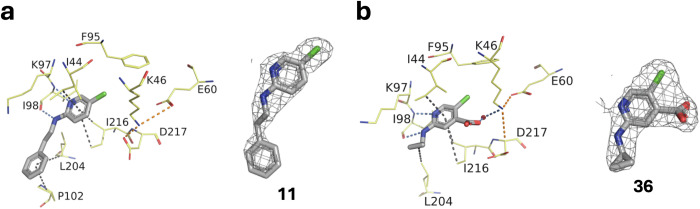
Structure of the selected derivatives of **F69**. Crystal structures of APH(2”)-IVa in complex with (**a**) **11** determined at 2.41 Å (PDB 9QNY) or (**b**) **36** determined at 2.30 Å (PDB 9QOE), and corresponding omit maps contoured at a sigma level of ± 1. Inhibitors are represented in gray sticks, residues involved in interactions are shown as yellow lines and the water molecule is represented as a red sphere. Interactions are shown as dashed lines: van der Waals interactions in gray, hydrogen bonds in blue and ionic bonds in orange.

We soaked these analogues on apo APH crystals to determine the structure of the complexes. We obtained the structure of six additional complexes, APH(2”)-IVa with **11**, **17**, **22**, **24**, **36**, and **68**. The three compounds substituted according to vector 2 and containing a sulfonamide (**17**, **22**, and **24**) were found in another binding site, in the C-terminal domain of the protein (not shown) and were set aside for the purpose of this study. The structures with **11** and **36** are shown in Fig. 3. The phenylethyl of **11** on vector 1 of **F69** established additional hydrophobic interactions with Pro102 and Leu204 (Fig. 3a), but this did not seem to improve inhibition: 57% for **11** compared with 68% for the parent molecule **F69**. Similarly, the cyclopropyl of **36** establishes a hydrophobic bond with Leu204. Even more interestingly, the carboxyl of **36** in vector 3 establishes a hydrogen bond with a water molecule, which itself interacts with the catalytic Lys46. This leads to a significant increase in inhibition to 87%. Structure of APH in complex with **68** is discussed later in the context of 7-azaindole optimization.

**Fig. 4:**
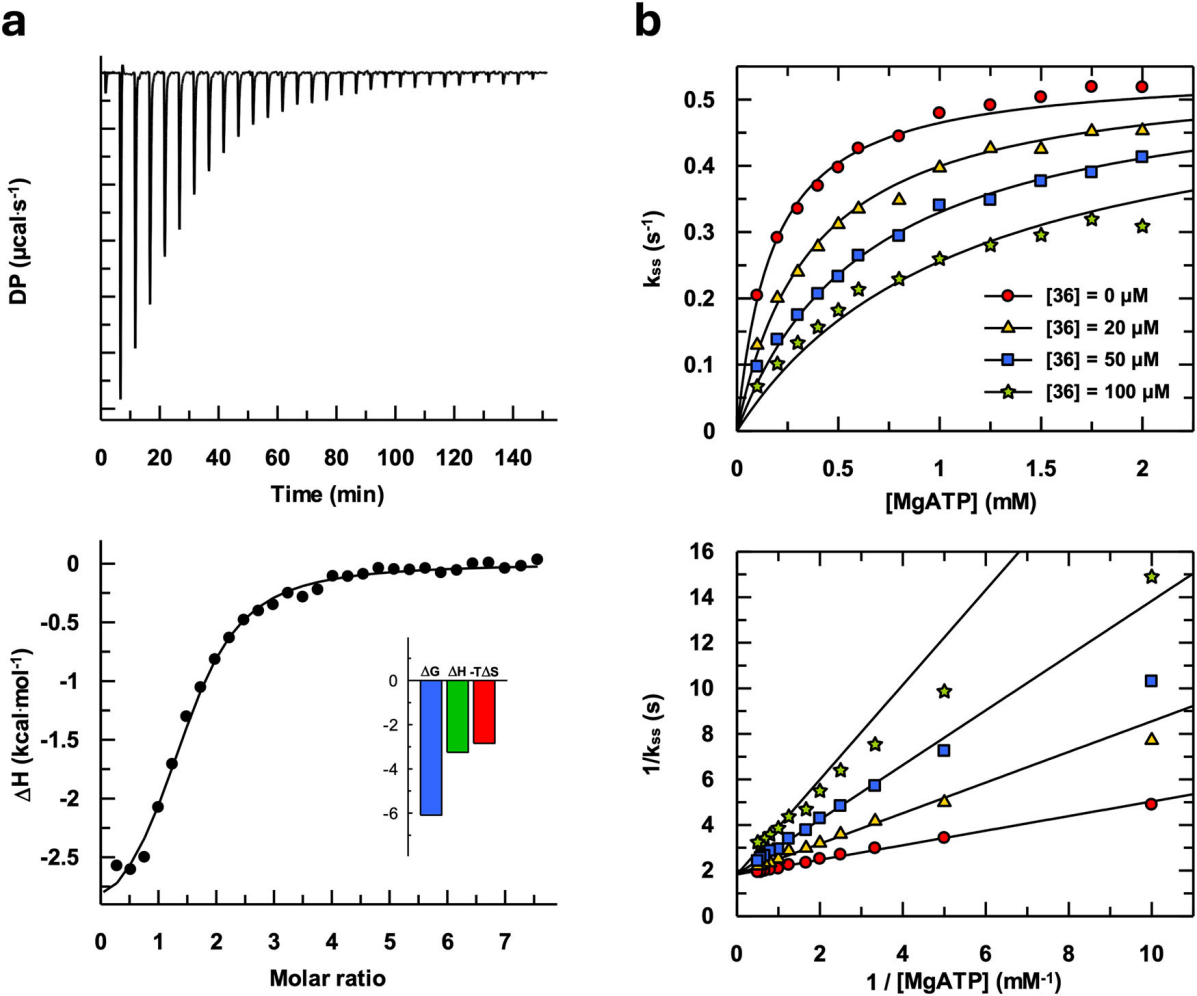
Affinity of APH(2”)-IVa for **36** and its ATP-competitive inhibition. **a** Differential heat released (top panel) and ITC binding curves (bottom panel) of **36** binding to APH(2”)-IVa. **b** Hyperbolic fitting of raw data (top panel) and Lineweaver-Burk representation (bottom panel) of the ATP competitive inhibition of APH(2”)-IVa by **36**. Final concentrations were 0.1 µM APH(2”)-IVa, 0-2 mM MgATP, 100 µM kanamycin A, 2 mM PEP, 140 µM NAD and 0-100 µM **36**. The red circles, yellow triangles, blue squares and green stars correspond respectively to 0, 20, 50 and 100 µM of **36**.

Given the strong inhibition observed with **36** at 500 µM, we decided to determine its affinity for APH(2”)-IVa and its inhibition constant. The affinity for **36** measured by Isothermal Titration Calorimetry (ITC) was relatively high for such a small molecule, with a dissociation constant (K_d_) of 37.8 µM (Fig. 4a). The binding was equally driven by enthalpy and entropy (inset of Fig. 4a), suggesting contributions of hydrogen bonds, van der Waals and hydrophobic interactions, and potentially conformational changes. Inhibition curves show that **36** acts as an ATP-competitive inhibitor of APH(2”)-IVa with an inhibition constant (K_i_) of 18.2 ± 1.2 µM (Fig. 4b).

**Fig. 5:**
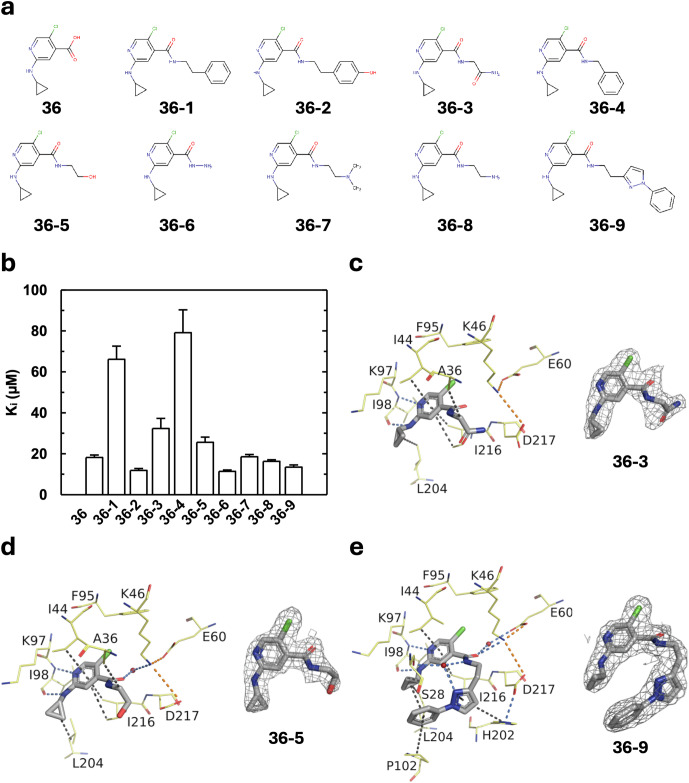
Characterization of derivatives of **36**. **a** Structure of the derivatives of **36** tested. (**b**) APH(2”)-IVa inhibition constants of **36** derivatives determined as in Fig. 4b. **c**–**e** Crystal structures and corresponding omit maps contoured at a sigma level of ± 1 of APH(2”)-IVa in complex with **36-3** determined at 2.11 Å (PDB 9QNX), **36-5** at 2.30 Å (PDB 9QP9) and **36-9** at 2.04 Å (PDB 9QPL). Inhibitors are represented in gray sticks, residues involved in interactions are shown as yellow lines and water molecules are represented as red spheres. Interactions are shown as dashed lines: van der Waals interactions in gray, hydrogen bonds in blue and ionic bonds in orange.

At this stage, we have ordered 9 analogues of **36** (Fig. 5a) and measured their K_i_ values (Fig. 5b). We succeeded in determining the structure of APH in complex with 3 analogues of **36** (Fig. 5c-e). The crystallographic structures of APH(2”)-IVa in complex with **36-3,** **36-5**, and **36-9** determined at 2.11, 2.30, and 2.04 Å, respectively, revealed interesting interactions with APH(2”)-IVa. First, it shows that an elongation of the arm at position 4 of the aminopyridine induces a conformational change of the catalytic aspartic acid 217, as in the case where a nucleotide is present in the binding pocket (PDB 4N57 and Fig. 5c–e). Second, in the case of **36-5** and **36-9**, the oxygen of the amide group at position 4 of the pyridine interacts with a water molecule, itself in interaction with Lys46 (Fig. 5d, e). Finally, in the case of **36-9**, the extension at position 4 adopts a conformation that brings the cycle closer to the cyclopropyl group, leading to a pseudo-macrocycle (Fig. 5e). This conformation is stabilized by several van der Waals interactions, involving the phenyl and cyclopropyl groups as well as Pro202. It also sequesters an additional water molecule, which interacts with the nitrogen atom of the amide function as well as Ser28.

However, although these analogues of **36** provide additional interactions with APH, the increase in inhibition is not significant in relation to the increase in size of the starting compound. This may be explained in part by the polarity of their sidechain (e.g.: **36-6** and **36-8**) or their intrinsic flexibility. Also, their common amide bond may not mimic well the acidic function of **36**.

### Extensions on vector 3 of 5-chloro-7-azaindole greatly increase the efficacy of APH inhibition

Based on previous results obtained with 2-amino-5-(chloro/bromo)pyridine, we ordered analogues of 5-chloro-7-azaindole, **2**, with substitutions at vectors 2 and 3 because they are expected to extend towards the catalytic residues.

**Fig. 6:**
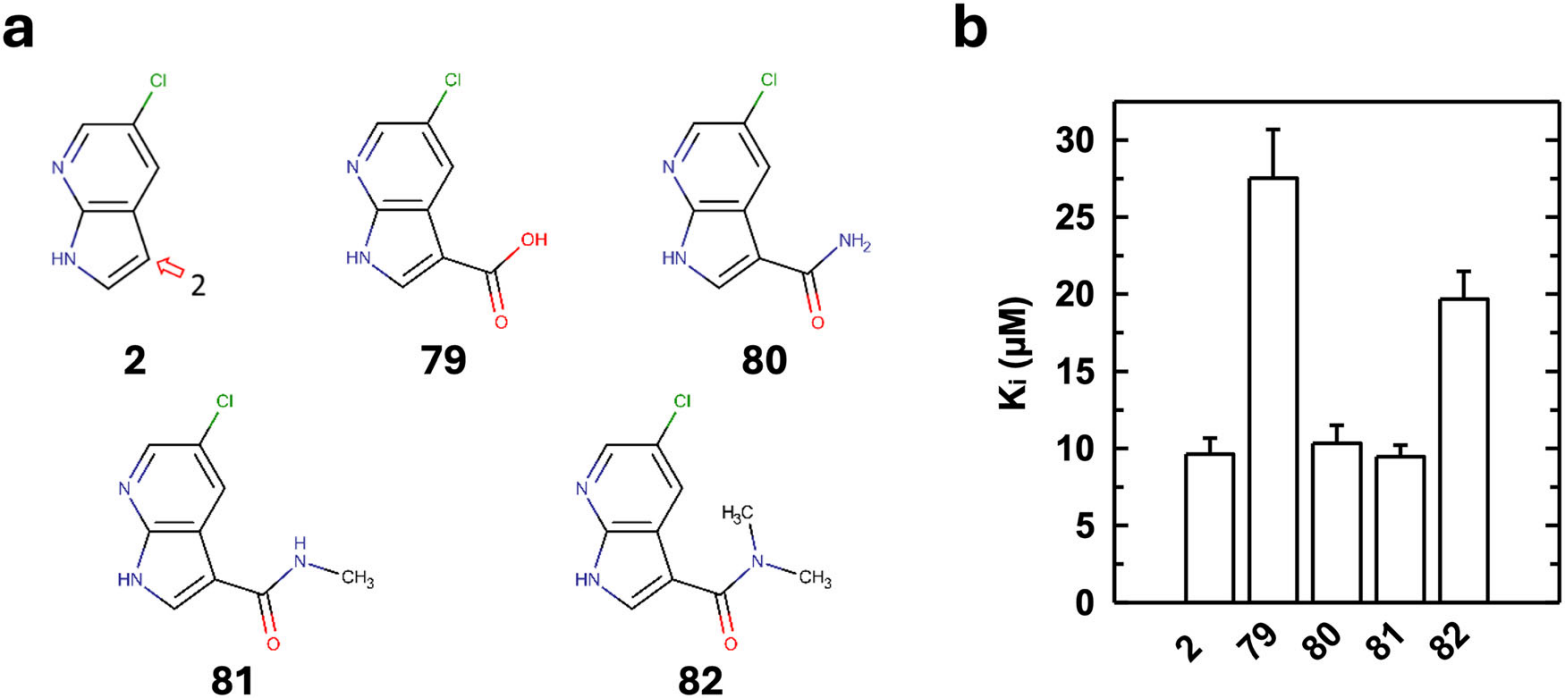
Characterization of azaindole derivatives at vector 2. **a** Structure of the more potent analogues of **2** at vector 2. **b** APH(2”)-IVa inhibition constants of the analogues of **2** determined as in Fig. 4b.

We selected 4 analogues of **2** at vector 2 based on inhibition assays at 500 µM (not shown). The chemical structures of these analogues are shown in Fig. 6a. Their K_i_ values were measured and are comparable to, or larger than, the K_i_ value of **2** (Fig. 6b).

**Fig. 7:**
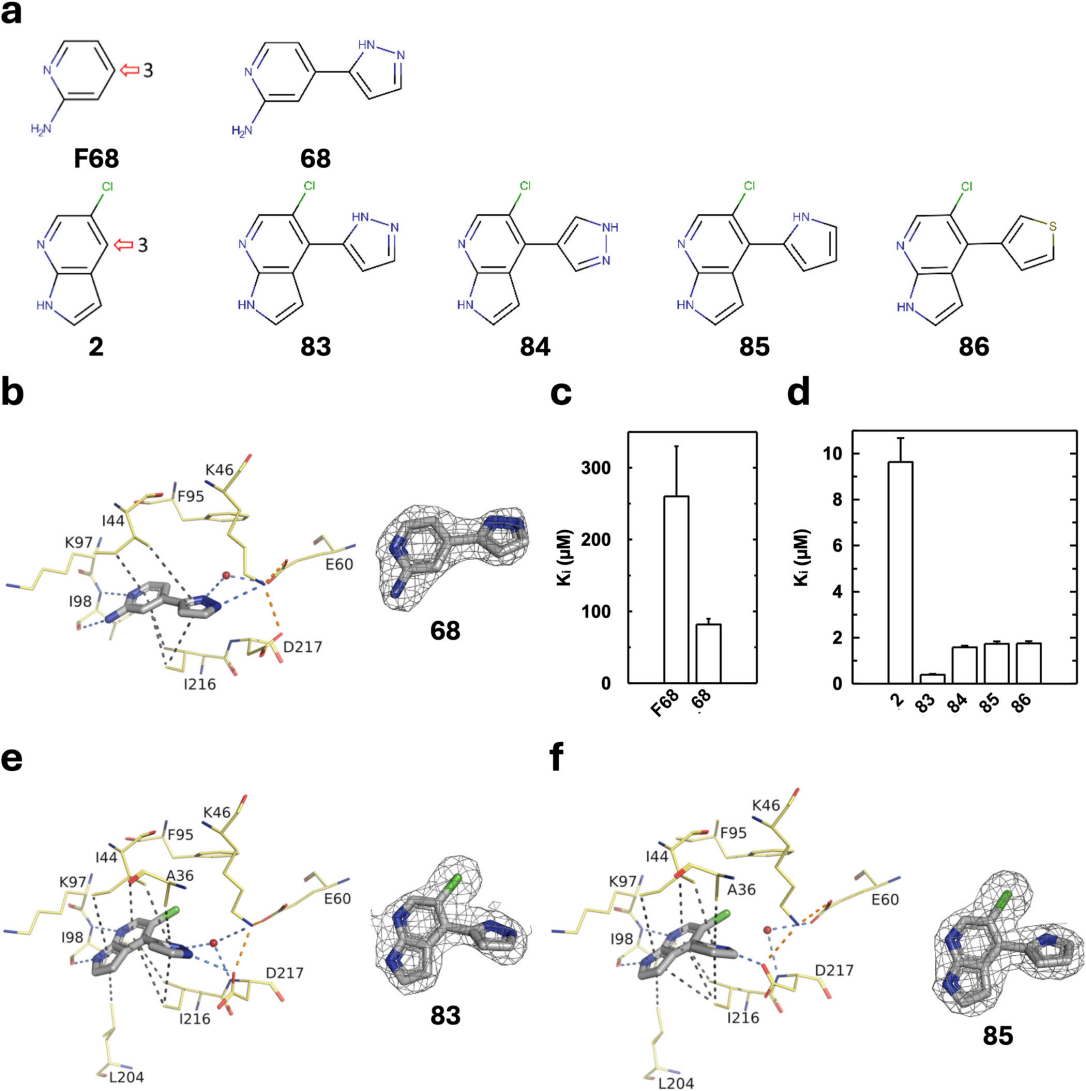
Characterization of the inhibitory effect and binding mode of 5-chloro-7-azaindole derivatives at vector 3. **a** Structure of the derivatives of 2-aminopyridine and 5-chloro-7-azaindole tested. **b** Crystal structures of APH(2”)-IVa in complex with **68** determined at 1.92 Å (PDB 9QOI) and corresponding omit map contoured at a sigma level of ± 1. (**c,** **d**) APH(2”)-IVa inhibition constants of analogues of (**c**) **F68** or (**d**) **2** determined as in Fig. 4b, except for **83**-**86** where their concentrations were decreased to 0-10 µM. **e**, **f** Crystal structures of APH(2”)-IVa in complex with **83** determined at 2.29 Å (PDB 9QOD) and **85** at 1.99 Å (PDB 9QOC), and corresponding omit maps. Inhibitors are represented in gray sticks, residues involved in interactions are shown as yellow lines and water molecules are represented as red spheres. Interactions are shown as dashed lines: van der Waals interactions in gray, hydrogen bonds in blue and ionic bonds in orange.

For the optimization of **2** in vector 3, we based our work on the structure of **68**. This molecule has, in addition to **F68**, a pyrazole group, which establishes a hydrogen bond with a water molecule and a direct interaction with the catalytic Lys46 (Fig. 7a, b). Also, despite the small size of **68**, it significantly inhibits APH (Fig. 7c). Furthermore, pyrazole stacks perfectly on Ile216. This prompted us to order a custom synthesis of **2** with a pyrazole, a pyrrole or a thiophene group in the corresponding position, i.e. at vector 3.

The addition of these groups at vector 3 of the 5-chloro-7-azaindole is highly favorable for APH(2”)-IVa inhibition (Fig. 7a, d). Compounds **83** to **86** exhibited much lower inhibition constant values than the parent compound, **2**. Compound **83** had a K_i_ of 0.39 ± 0.04 µM while **84** had a K_i_ of 1.58 ± 0.07 µM, suggesting that the position of the nitrogen atoms in the pyrazole group is important.

From a structural point of view, the efficiency of **83** to inhibit APH activity can be explained by the interaction of one nitrogen of the pyrazole group with a sequestered water molecule, which establishes a network of interactions with two residues important for the catalysis, Lys46 and Asp217 (Fig. 7e). Additionally, the other nitrogen of the pyrazole makes a direct interaction with Asp217, which is thus tilted toward the hinge. The single nitrogen atom of the pyrrole group in **85** interacts only with Asp217 and not with the water molecule, as shown by the crystal structures of this compound in APH(2”)-IVa (Fig. 7f).

### Characterization of the hit compound 83 on different APHs

**Fig. 8:**
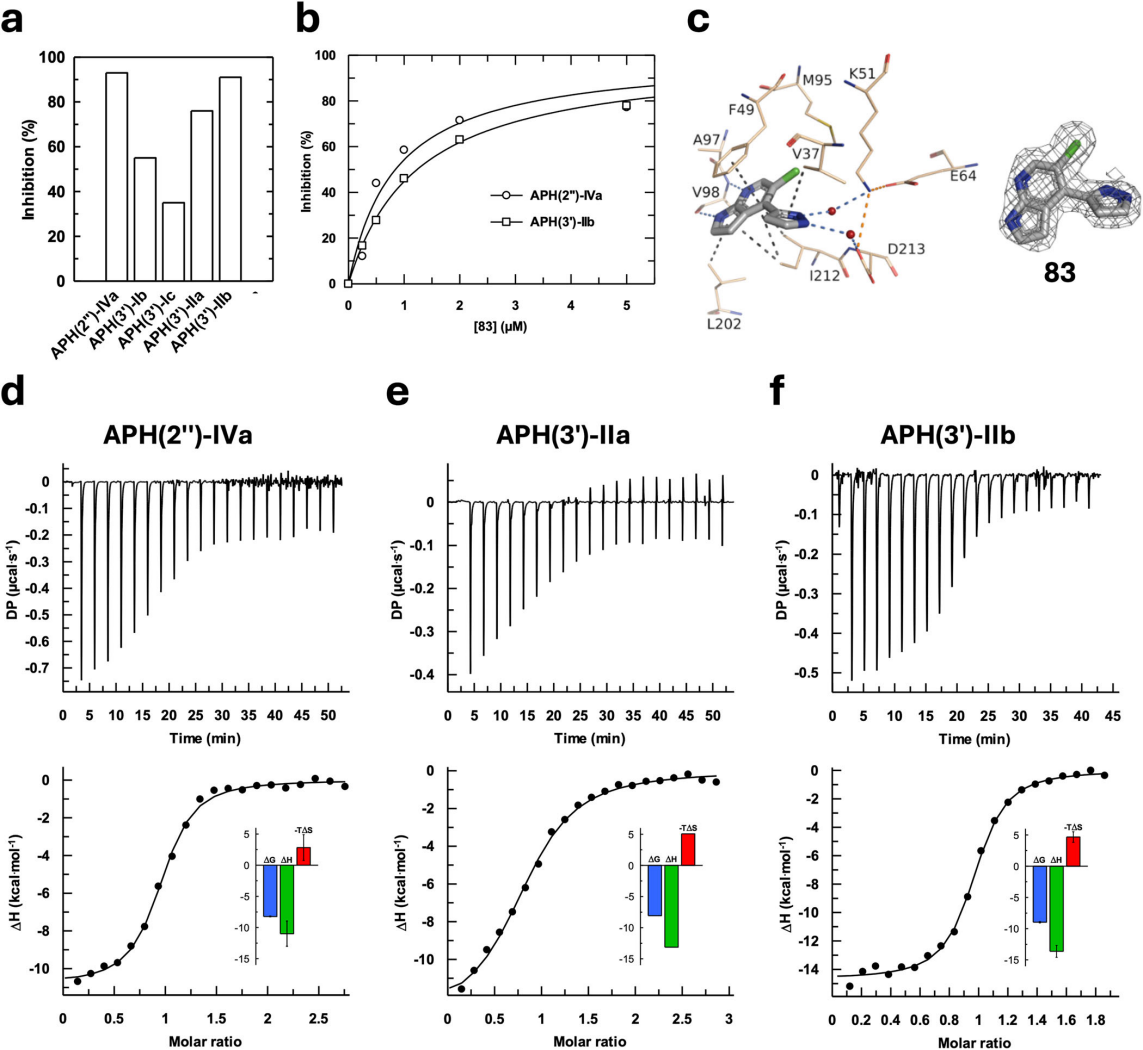
Characterization of the effect of **83** on the activity of several APHs. **a** Inhibition of the activity of APH(2”)-IVa, APH(3’)-Ib, APH(3’)-Ic, APH(3’)-IIa and APH(3’)-IIb by 100 µM of **83**. **b** IC_50_ of **83** with APH(2”)-IVa from *E. casseliflavus* or APH(3’)-IIb from *P. aeruginosa* determined by HPLC. **c** Crystal structure of APH(3’)-IIb in complex with **83** determined at 1.97 Å (PDB 9QOS) and corresponding omit map contoured at a sigma level of ± 1. Inhibitor is represented in gray sticks, residues involved in interactions as beige lines and water molecules are represented as red spheres. Interactions are shown as dashed lines: van der Waals interactions in gray, hydrogen bonds in blue and ionic bonds in orange. **d**–**f** ITC curves and thermodynamic profiles of binding of **83** to APH(2”)-IVa, APH(3’)-IIa and APH(3’)-IIb, respectively.

At this stage, **83** being the most potent inhibitor identified so far, we decided to characterize its effect in more detail. To do so, we first measured its inhibitory effect on four other APH(3’)s from pathogens of the WHO Priority-1’s list^24^: APH(3’)-Ib from *E. coli* and *K. pneumoniae*, APH(3’)-Ic from *A. baumannii*, APH(3’)-IIa from *K. pneumoniae* and APH(3’)-IIb from *P. aeruginosa* (Fig. 8a). We measured moderate levels of inhibition with APH(3’)-Ib and APH(3’)-Ic (55 and 35%, respectively) and higher levels of inhibition with APH(2”)-IVa, APH(3’)-IIa and APH(3’)-IIb (95, 76 and 91%, respectively). **83** has a relatively low IC_50_ for APH(3’)-IIb, with a value of IC_50_ of 1.23 ± 0.04 µM comparable to that of 0.84 ± 0.13 µM for APH(2”)-IVa (Fig. 8b). The crystal structure of APH(2”)-IVa in complex with **83** has been determined and discussed above (Fig. 7e). We were also able to determine the structure of **83** in complex with APH(3’)-IIb. The compound shows a similar binding mode in the nucleotide binding site of the two APHs with the same nature of interactions between the protein and **83** (Fig. 8c). Interestingly, it differs from the binding mode in the mammalian protein kinase Erk-2 (PDB 9QQJ, see accompanying manuscript^25^).

The affinities of **83** for APH(2”)-IVa, APH(3’)-IIa and APH(3’)-IIb were measured by ITC (Fig. 8d–f). The experiments revealed a similarly high affinity for these enzymes, with K_d_ values of 1.06 ± 0.17 µM (n = 3) for APH(2”)-IVa, 1.25 µM (n = 1) for APH(3’)-IIa and 0.27 ± 0.05 µM (n = 2) for APH(3’)-IIb. These values are very close to the IC_50_ values measured (Fig. 8b). All interactions show similar energetically favorable and enthalpy-driven thermodynamic profiles, with ΔH values of -11.0, -13.1, and -13.6 kcal.mol^-1^ for APH(2”)-IVa, APH(3’)-IIa and APH(3’)-IIb, respectively (Fig. 8d–f). These results suggest that in all these APHs, interactions with **83** are characterized by hydrogen bonds and van der Waals interactions, as confirmed by our structures.

### Hit compound makes clinical bacterial isolates more sensitive to kanamycin A

To test whether **83** is effective in increasing aminoglycoside sensitivity in bacteria, we performed checkerboard assays in the presence of **83** on several isolates from two hospitals (Hamilton General Hospital and Nîmes Hospital). We used isolates of *P. aeruginosa* or *S. aureus* expressing different APHs. We began by measuring the minimal inhibitory concentrations (MICs) of these strains for several aminoglycosides and carbapenems (Supplementary Table 6). We then measured these MICs at different concentrations of **83** from 0 to 128 µg mL^−1^ (585 µM), in the presence or absence of a sub-inhibitory concentration of imipenem (Table 3 and Supplementary Fig. 3). In all cases, we found that **83** alone, i.e. in the absence of antibiotics, had no impact on bacterial growth.

**Table 3:** Effect of **83** on the MIC of kanamycin A on different bacteria

	MIC of kanamycin A (µg mL^−1^)		
[83] (µg mL^−1^)	*P. aeruginosa* C0307 0 µg mL^−1^ imipenem	*P. aeruginosa* C0307 0.05 µg mL^−1^ imipenem	*S. aureus* C0032 0.125 µg mL^−1^ imipenem
0	64	64	4096
2	64	64	4096
4	64	64	4096
8	64	64	4096
16	64	64	4096
32	64	64	2048
64	64	64	2048
128	32	32	2048

MIC of kanamycin A was estimated after 18 h at 37 °C on *P. aeruginosa* C0307 in the absence or in the presence of 0.05 µg mL^−1^ of imipenem or on *S. aureus* C0032 in the presence of 0.125 µg mL^−1^ of imipenem.

*P. aeruginosa* strain C0307, expressing APH(3’)-IIb, showed resistance to kanamycin A with an MIC of 64–128 µg mL^−1^. When this strain was treated with **83** in combination with kanamycin A, the MIC value decreased, demonstrating the compound’s ability to reduce the concentration of kanamycin A required to inhibit the growth of this strain. At a concentration of 128 µg mL^−1^ of **83**, the MIC of kanamycin A was decreased to 32 µg mL^−1^ (Table 3 and Supplementary Fig. 3a). These results show that **83** is capable of slightly increasing the sensitivity of this resistant clinical isolate of *P. aeruginosa* to kanamycin A.

We tried using sub-inhibitory concentrations of different carbapenems to weaken the bacterial cell wall, but this did not impact the effect of **83** on *P. aeruginosa* strain C0307 (Table 3 and Supplementary Fig. 3b).

*S. aureus* strain C0032 expresses APH(3’)-IIIa and shows a high level of resistance to kanamycin A (MIC of 4096 µg mL^−1^). It was tested in the presence of a sub-inhibitory concentration of imipenem to weaken the bacterial cell wall, in addition to treatment with **83** and kanamycin A. Under these conditions, the MIC value was also reduced in the presence of **83**. At 32 µg mL^−1^ of **83**, the MIC was reduced to 2048 µg mL^−1^ of kanamycin A (Table 3 and Supplementary Fig. 3c). These results show that **83** is capable of slightly improving the sensitivity of this resistant strain of *S. aureus* to kanamycin A. However, the MIC values are still very high, and further optimization of this hit compound will be necessary.

**Fig. 9:**
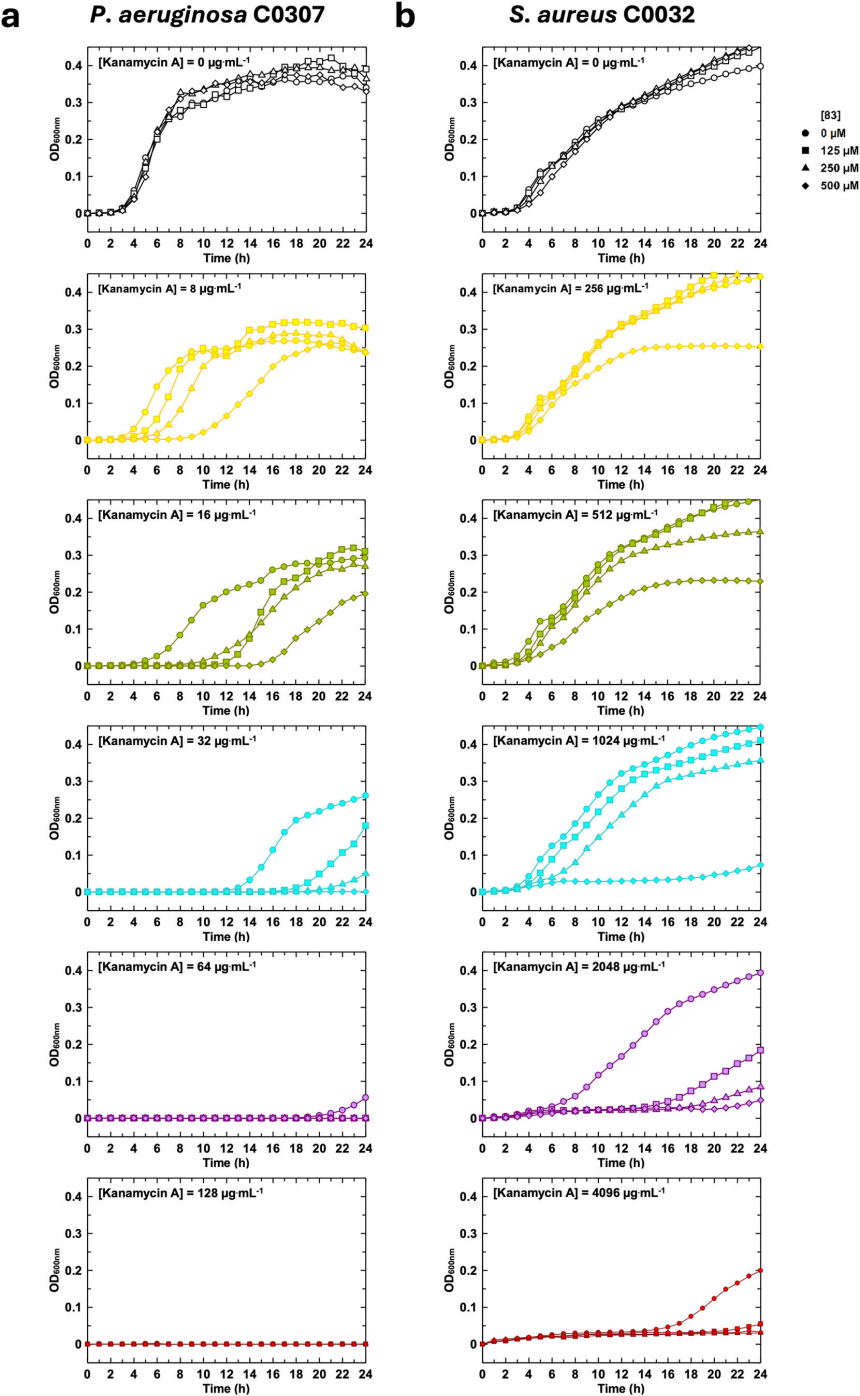
Characterization of the effect of **83** on bacterial growth kinetics. Measurement of the 24-h growth kinetics of (**a**) *P. aeruginosa* C0307 or (**b**) *S. aureus* C0032 in the presence of increasing concentrations of kanamycin A and different concentrations of **83**. The characteristics of these strains are shown in the Supplementary Table 6. The symbols circles, squares, triangles and diamonds correspond respectively to 0, 125, 250 and 500 µM of **83**. The colors black, yellow, green, cyan, mauve and red correspond respectively to 0, 8, 16, 32, 64 and 128 µg mL^−1^ of Kanamycin A in (**a**) and 0, 256, 512, 1024, 2048 and 4096 µg mL^−1^ of Kanamycin A in (**b**).

We then measured the growth kinetics of these bacteria in the presence of different concentrations of kanamycin A and **83** over 24 h (Fig. 9 and Supplementary Fig. 4). Only the curves at the six most representative kanamycin A concentrations are shown. We have excluded concentrations of kanamycin A where there was no growth, and those where no **83** effect was observed (except the 0 µM kanamycin A). Again, no toxicity of **83** alone was observed on *P. aeruginosa* or *S. aureus* up to 500 µM (109 µg mL^−1^).

In *P. aeruginosa* strain C0307, a significant **83** concentration-dependent growth retardation was observed (Fig. 9a). At 8 µg mL^−1^ kanamycin A, **83** at 500 µM delayed the onset of growth by around 6 h, and growth kinetics in the exponential phase were also affected. At 16 µg mL^−1^ kanamycin A, the effect is even more pronounced, with latency extended by 12 h in the presence of 500 µM **83**. At this **83** concentration and at 32 µg mL^−1^ kanamycin A, growth did not begin after 24 h of incubation. Other *P. aeruginosa* isolates possessing APH(3’)-IIb showed similar effects (Supplementary Fig. 4).

The same applies to *S. aureus* C0032 strain, which possesses an APH(3’)-IIIa and is highly resistant to kanamycin A (Fig. 9b). In the absence of imipenem and in the presence of 256 and 512 µg mL^−1^ kanamycin A, 500 µM **83** slowed growth kinetics and saturation appeared to occur at lower optical density at 600 nm (OD_600nm_). At 1024 and 2048 µg mL^−1^ kanamycin A, 500 µM **83** increased latency by 14 and 18 h, respectively. Finally, at 4096 µg mL^−1^ kanamycin A, while in the absence of **83** the bacteria started to grow at 14 h, no growth was detected at 250 and 500 µM **83** after 24 h.

In conclusion, **83** is capable of slowing the growth of aminoglycoside-resistant bacteria in a dose-dependent manner. The effect on MICs remains low, suggesting that optimization is still needed to compete effectively with ATP, which is present at millimolar concentrations in bacteria.

## Discussion

While new APHs are still being discovered regularly (for example, Lund et al.^26^), the design of specific inhibitors of these enzymes could provide a means of combating bacterial resistance to aminoglycosides. Using an FBDD approach, we identified a hit compound, **83**, capable of inhibiting several APHs found in human pathogens, including Gram-negative bacteria of the 2024 WHO bacterial priority pathogens list. In vitro, on purified enzymes, **83** acts as a potent and competitive inhibitor of ATP: the K_d_ values we measured are 40 to 100-fold lower than the reported Michaelis constant (K_m_) values for ATP (101 µM for APH(2”)-IVa^27^, 45 µM for APH(3’)-IIa^28^ and 24 µM for APH(3’)-IIb^29^).

The structural similarity of the nucleotide-binding site between APHs and eukaryotic protein kinases (ePKs) had already motivated several research teams to test the effect of ePK inhibitors on APHs. Thus, Daigle et al.^12^ examined the effect of known ePK inhibitors of the class of indole carbazoles, flavanoids and isoquinoline sulfonamides on two aminoglycoside kinases, APH(3’)-IIIa found in *S. aureus* and *Enterococcus spp*. and AAC(6’)-APH(2”) from *S. aureus*, *E. coli* and *E. faecium*. Isoquinoline sulfonamides proved the most effective, with inhibition constants of several tens of micromolar. However, these compounds were unable to reverse antibiotic resistance in bacterial cultures expressing APH.

**Fig. 10:**
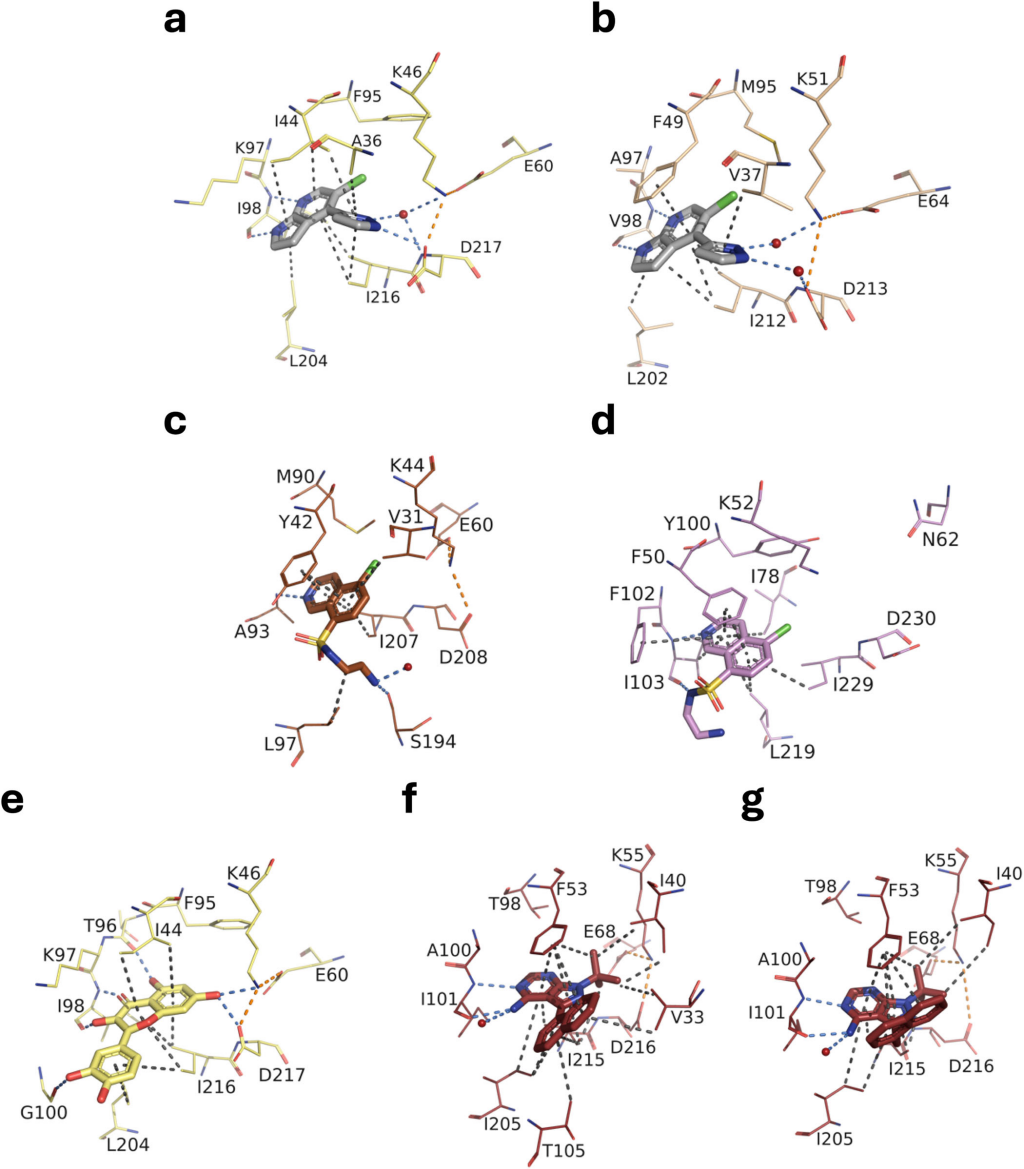
Comparison of the structures of APH-competitive nucleotide inhibitor complexes. **a**, **b** Structures of **83** in complex with (**a**) APH(2”)-IVa (PDB 9QOD) and (**b**) APH(3’)-IIb (PDB 9QOS). **c**, **d** Structures of CKI-7 in complex with (**c**) APH(3’)-IIIa (PDB 3Q2J) and (**d**) APH(9)-Ia (PDB 3Q2M). **e** Structure of quercetin in complex with APH(2”)-IVa (PDB 4DFU). **f,** **g** Structures of (**f**) 1-NA-PP1 (PDB 4GKH) and (**g**) 1-NM-PP (PDB 4GKI) in complex with APH(3’)-Ia. Inhibitors are represented as sticks, residues involved in interactions are shown as lines: yellow for APH(2”)-Iva (**a**, **e**), beige for APH(3’)-IIb (**b**), brown for APH(3’)-IIIa (**c**), mauve for APH(9)-Ia (**d**) and red for APH(3’)-Ia (**f, g**). Water molecules are represented as red spheres. Interactions are shown as dashed lines: van der Waals interactions in gray, hydrogen bonds in blue and ionic bonds in orange.

Fong et al. succeeded in determining the structures of APH(3’)-IIIa (PDB 3Q2J) and of the atypical APH(9)-Ia from *L. pneumophila* (PDB 3Q2M) in complex with CKI-7, a casein kinase 1 inhibitor, capable of inhibiting these APHs with inhibition constants of 66 and 159 µM, respectively^14,30^. The position of CKI-7 isoquinoline in the APH(3’)-IIIa and APH(9)-Ia sites is similar to that of **83** in the APH(2”)-IVa and APH(3’)-IIb (Fig. 10a–d). The aromatic ring stacks against hydrophobic residues, and the heterocycle nitrogen makes a hydrogen bond with a backbone atom of a hinge residue. The chlorine is pointing into the pocket facing the gatekeeper residue. The remainder of the CKI-7 molecule, its aminoethylsulfonamide group, adopts a different configuration in the two APHs and does not participate in major interactions with the protein, confirming our observation that the extension at vector 1 of **83** is not advantageous.

Following a screening of 80 ePKs on 12 different APHs, Shakya *et al*. identified the flavonol quercetin as the best candidate for reversing APH-mediated aminoglycoside resistance. Quercetin inhibited all the APHs tested with K_i_ values ranging from 0.07 to 25 µM^31^. The crystallographic structure of quercetin in complex with APH(2”)-IVa (PDB 4DFU) reveals a position of the B ring similar to our substituents at vector 3 of 5-chloro-7-azaindole. Interestingly, the 7-hydroxyl of quercetin formed hydrogen bonds to the side chains of two catalytic residues, Lys46 and Asp217 (Fig. 10e). In our structures of APHs in complex with **83**, these interactions occur via a water molecule. This suggests that the introduction of a hydroxyl group on the pyrazole could be of interest. This is described in the accompanying manuscript^25^.

Other ePK inhibitors, such as pyrazolopyrimidines, were co-crystallized with APH(3’)-Ia from *E. coli* and show interactions similar to the nucleobase^31^ (PDB 4GKH and 4GKI, Fig. 10f-g). Modifications at position N1, C3 and N4 of pyrazolopyrimidine did not increase the in vitro APH inhibition efficacy, further confirming our observations. However, it is interesting to note that two molecules, 1-NA-PP1 and 1-NM-PP1, substituted in C3 and inhibiting APH(3’)-Ia with an inhibition constant of 21.5 and 34.4 µM, respectively, demonstrated rescue activity of kanamycin A on the resistant hyper-permeable efflux mutant of *E. coli* (Δ*tolC* Δ*bamB*).

In another study, Boehr et al.^32^ tested the inhibition of APHs by the lipid kinase inhibitor wortmannin. Wortmannin was able to inhibit APH(2”)-Ib (90% inhibition at 1 mM), but did not affect APH(3’)-IIIa.

Finally, another way of specifically inhibiting APHs would be to find non-competitive ATP inhibitors. Three studies have reported such inhibitors, but none were able to effectively inhibit multiple APHs, thus reducing their therapeutic potential^33–35^.

Compared with all other APH inhibitors reported in the literature, our hit compound is the only one that is ATP-competitive but not a known protein kinase inhibitor. Its specificity toward APHs and not for human kinases is described in the accompanying manuscript^25^. On the other hand, it is one of the smallest and the most effective, with promising inhibition constants close to 1 µM on several APHs. In addition, our hit compound is capable of slowing the growth of clinical isolates of *P. aeruginosa* and *S. aureus* expressing one or more APHs. In the accompanying manuscript^25^, we propose chemical modifications of this hit compound to increase its efficacy and bioavailability in bacteria, while maintaining its low toxicity for human cells.

## Methods

### Molecular biology

*E. coli* DH5α and BL21 strains have been used for plasmid production and protein expression, respectively. The *aph(2”)-IVa* and *aph(3’)-IIb* genes were ordered from Integrated DNA Technologies™. In-fusion cloning was performed to insert the genes into the plasmid pET-15b. The In-Fusion cloning product was used to transform both *E. coli* DH5α and BL21. Bacterial glycerol stocks were produced after plasmid extraction (Macherey-Nagel Plasmid kit) and sequencing control (Eurofins). Sequencing revealed that, in the case of *aph(3’)-IIb*, a spontaneous mutation occurred, leading to a M95L mutant which has been characterized elsewhere and shows similar substrate affinities to the wild-type APH(3’)-IIb^29^.

### Protein expression and purification

APHs were produced in *E. coli* BL21 and purified as already described^29,35^. Briefly, protein expression was induced by adding 1 mM isopropyl β-D-1-thiogalactopyranoside (IPTG) in a 2 L culture in LB at OD_600_ between 0.6 and 0.8 and incubation overnight at 18 °C. The bacterial pellets were then resuspended in 100 mL of Buffer A (50 mM NaH_2_PO_4_, 300 mM NaCl, 10 mM Imidazole, 1 mM dithiothreitol (DTT), pH 8) supplemented with two tablets of cOmplete™ protease inhibitor cocktail (Roche), 10 µg mL^−1^ of Lysozyme (Roche) and 1 µg mL^−1^ of DNase (Roche). The resuspended pellets were pooled and sonicated on ice for 10 min using 2-second pulses. The supernatant was then filtered on 5 µm followed by 0.45 µm filters before the affinity chromatography using two 5 mL HisTrap columns (Cytiva) connected in tandem on an Aktä Pure. Elution of the protein was performed using a linear gradient to 50% of Buffer B (50 mM NaH_2_PO_4_, 300 mM NaCl, 500 mM Imidazole, 1 mM DTT, pH 8). The proteins were eluted at approximately 12% of Buffer B and collected in fractions of 2 mL. Fractions of interest were pooled, concentrated, and injected on a 26/60 Superdex™ 75 prep grade (GE Healthcare) size exclusion column. Two buffers were used depending on the experiments performed: Buffer Tris (50 mM Tris, 40 mM KCl, 1 mM MgCl_2_, 1 mM DTT, pH 7.5) for enzymatic measurements and TSA or Buffer HEPES (50 mM HEPES, 40 mM KCl, 1 mM MgCl_2_, 1 mM DTT, pH 7.5) for ITC experiments. Proteins eluted in a single peak, and the corresponding fractions were pooled and concentrated to 10–20 mg mL^−1^, aliquoted, and stored at −80 °C.

### Chemicals

All chemicals were purchased from Sigma-Aldrich, BLD Pharm, Enamine or AGV Discovery.

### Thermal shift assays

Thermal shift assays were performed using an RTqPCR machine Mx3005P (Stratagene) in 96-well plates (Thermo-Fast low Profile, ThermoScientific) by mixing the protein at 5 µM, SYPRO® Orange (Sigma Aldrich) at 5000×, and fragments at 500 µM. Fluorescence was followed with Cy3 and SYAL filters. Denaturation curves were collected using MxPro-Mx3005P v4.10 software and analyzed using the implemented version of w*TSA-CRAFT*^36^.

### Kinetic measurements using a coupled enzyme system assay

Enzymatic reactions were carried out as previously described^34^ at 25 °C in 96 half-well flat-bottom plates (Corning) in a reaction volume of 100 µL in Buffer Tris. In the inhibition assays, final concentrations were 0.1 µM APH(2”)-IVa, 350 µM MgATP, 100 µM kanamycin A, 500 µM fragments, 2 mM phosphoenol pyruvate (PEP), 140 µM reduced β-nicotinamide adenine dinucleotide (NADH) and 1× lactate dehydrogenase/pyruvate kinase (LDH/PK). For K_i_ determination, final concentrations are given in the figure captions. Plates were placed in a Clariostar plate reader. Reactions were initiated by the addition of MgATP, and the measured absorbance at 340 nm (CLARIOstar v. 5.40 R2 and MARS v. 3.31, BMG Labtech) was divided by the extinction coefficient of NADH (6.22 mM^−1^ cm^−1^). The initial rate constants (k_ss_) were then measured using a linear regression of the absorbance as a function of time using GraFit software (v. 7.0.3, Erithacus software). Inhibition constants were determined by global fittings of the dependences of k_ss_ as a function of ATP and inhibitor concentrations, using a competitive inhibition equation and GraFit software. The standard error of the K_i_ value was determined in the GraFit software based on the global fitting of four experimental series.

### IC_50_ measurements using quantification of ATP and adenosine diphosphate (ADP) by HPLC

This method is a direct measurement of the amount of ADP produced during APH enzymatic reaction, as already described^35,37^. It is more sensitive than the coupled enzyme system assay, which is worthwhile for experiments with APH(3’)-IIb because of the low concentration of ATP used with this enzyme, close to its K_m_ value. The reaction mixture consists of the APH and its substrates, as well as a potential inhibitor for inhibition measurements. Reactions were initiated by adding the ATP solution supplemented or not with the inhibitor, in a thermostated beaker containing the APH. For experiments with APH(2”)-IVa, final concentrations were 0.1 µM APH, 350 µM MgATP, 100 µM kanamycin A and 0–5 µM inhibitor. For experiments with APH(3’)-IIb, final concentrations were 25 nM APH, 15 µM MgATP, 100 µM kanamycin A and 0–5 µM inhibitor. The reaction was stopped at several time points by adding 80 µL of the enzymatic reaction mixture from the beaker to 40 µL of 10% perchloric acid (PCA) in an Eppendorf tube. Samples were then centrifuged for 30 min at 12750 rpm and 8 °C. 100 µL of the supernatant was then mixed with 900 µL of mobile phase (250 mM NaH_2_PO_4_, 50% Acetonitrile, pH 5.5) supplemented with KOH to readjust the pH to 5.5 before injection. Sample vials were loaded into the autosampler of an Agilent 1260 Infinity HPLC system. 100 µL of samples were injected on a SAX PartiSphere column (AIT France) at a flow rate of 1 mL min^−1^. ATP and ADP peaks were integrated, and the area under the peaks was calculated using OpenLab CDS software (v. ChemStation, Agilent). The k_ss_ values were then measured using linear regression in GraFit software and IC_50_ determined by fitting with a hyperbola the percentage of inhibition as a function of inhibitor concentration. The standard error of the IC_50_ value was determined in the GraFit software based on the fitting of the experimental data.

### Affinity measurement by Isothermal Titration Calorimetry

ITC experiments were performed on three different machines: a MicroCal VP-ITC (GE Healthcare), a MicroCal PEAQ-ITC (Malvern), or a MicroCal iTC200 (Malvern). The architecture and principles are similar for the three different systems. The experiments were carried out in Buffer HEPES at 25 °C. Titrations consisted of twenty injections of 1.5 µL inhibitors, every 120 seconds, into the measuring cell using the injection syringe rotating at 750 rpm. The data were analyzed using the MicroCal PEAQ-ITC software (v. 1.52, Malvern) using the single-site binding model to fit the raw data. The standard error of the K_d_ value was determined from measurements made on distinct samples (n indicated in the text).

### X-ray crystallography and structure refinement

Crystallogenesis of APH(2”)-IVa and APH(3’)-IIb has been carried out as described previously^29,35,37^. We soaked the crystals of the apo forms by introducing ligands in a clear drop consisting of 2 µL of the reservoir next to the drops containing crystals. This new drop was then merged with the drop containing crystals. The ligands were used at a final concentration of 5 mM by adding 0.2 µL of a 100 mM stock solution. Soaking times varied from a few seconds to 1 h, depending on the behavior of the crystal. The crystals were frozen in liquid nitrogen before being shipped for diffraction at the European synchrotron radiation facility (ESRF, France) or Alba (Spain). Structures were solved by molecular replacement with Phaser-MR, using a previously solved structure of the protein in complex with ADP (PDB 4N57) as a model after removal of ligand and water molecules. The structures were subsequently built and refined using Coot^38^ (v. 0.9.4) and Phenix^39^ (v. 1.19.2). X-ray data and refinement statistics are given in the Supplementary Table 1-5.

### MIC measurements and checkerboard assays

Antibiotic susceptibility of bacteria has been tested for various clinical strains listed in the Supplementary Table 6. These strains are from the microbiological collection of Nîmes University Hospital, France (NH) or Hamilton General Hospital, ON, Canada (HGH). They are available upon direct request from the relevant hospitals for academic research purposes (through an MTA) and are disposed of after use. MICs of several antibiotics were characterized using the broth micro-dilution method in 96-well microtest plates (Starstedt). First, glycerol stocks were scraped and streaked onto Brain Heart Infusion (BHI) agar plates for clinical strains. Plates were then incubated overnight in a static incubator at 37 °C. On the second day, several clones were collected and used to prepare an inoculum with an OD_600nm_ of between 0.08 and 0.1 in a saline solution. The inoculum was diluted 1:200 in cation-adjusted Müller-Hinton broth (CAMHB).

The final volume of reaction was 100 µL, and serial dilutions of antibiotics were made from column 1 to 10 for a final concentration of antibiotics from 1 to 512 µg mL^−1^. Columns 11 and 12 were retained for positive and negative controls, respectively. Inocula were added to columns 1 to 11, and CAMHB was added to the negative control column. The final OD_600nm_ of the inocula in the plates was contained between 0.0008 and 0.001. Plates were incubated at 37 °C for 16 to 20 h, except for enterococci strains, for which plates were incubated for 24 h. Plates were placed in a plate reader (Tecan) after incubation, and MIC values were determined as the lowest concentration of aminoglycoside resulting in complete inhibition of bacterial growth as assessed by reading OD_600nm_ after 18 h of incubation (Magellan v. 6.2 software, Tecan).

Checkerboard assays were performed in 96-well microtest plates (Starstedt) with CAMHB complemented with dimethyl sulfoxide (DMSO), depending on the percentage of DMSO with the ligand used. Inocula were prepared as described above for MIC measurements. The aminoglycoside concentration was varied from row A to H, and the concentration of the potential inhibitor tested was varied from column 1 to 9. If the test is performed in the presence of imipenem, the latter is present in columns 1 to 9, and an additional column is used to test the condition without imipenem (column 10).

### Bacterial growth kinetics

*P. aeruginosa* was pre-cultured in Mueller-Hinton (MH) broth at 37 °C under agitation overnight. The optical density at 600 nm (OD_600nm_) was then measured to adjust the bacterial concentration. An initial suspension was prepared at an OD_600nm_ of 0.1 (≈1 × 10⁷ colony forming units mL^−1^) and further diluted to obtain a final working concentration of 5 × 10⁵ colony forming units mL^−1^.

Test compounds were initially dissolved at 100 mM in 100% DMSO and serially diluted to obtain a 2 mM intermediate stock solution in 4% DMSO. Working concentrations (125, 250, and 500 µM) were then prepared by additional twofold dilutions in the same solvent. Finally, 25 µL of each solution was added to the wells, yielding final concentrations of 0, 125, 250, and 500 µM with a DMSO concentration of 1%. Kanamycin A was prepared as an 8192 µg mL^−1^ stock solution in sterile water and serially diluted two-fold. For each condition, 50 µL of the diluted solution was added to the wells. Final concentrations ranged from 8 to 4096 µg mL^−1^.

Bacterial suspensions were added to a 96-well plate in a final volume of 100 µL per well with the following setup. Column 1 was the negative control (MH medium + compound, no bacteria). Column 2 was the positive control (MH medium + compound + bacteria, no antibiotic). Columns 3 to 12 contained antibiotics with decreasing concentrations of kanamycin A (4096 to 8 µg mL^−1^). Plates were incubated at 37 °C for 24 h, and OD_600nm_ was measured every hour using an Infinite M Nano absorbance reader (Tecan, Männedorf, Switzerland).

Each test compound was evaluated at four concentrations (0, 125, 250, and 500 µM), with experimental duplicates performed across two rows. Analysis and representations were conducted using GraFit.

### Reporting summary

Further information on research design is available in the Nature Portfolio Reporting Summary linked to this article.

## Supplementary information

Description of Additional Supplementary Files

Supplementary Information

Supplementary data

Reporting Summary

## Data Availability

The authors declare that the data supporting the findings of this study are available within the paper and in Supplementary Data. Should any raw data files be needed in another format, they are available from the corresponding author upon reasonable request. All structural data that support the findings of this study have been deposited in the Protein Data Bank (https://rcsb.org), with the accession codes: 9QMR, 9QN6, 9QPD, 9QNS, 9QNN, 9QNQ, 9QNW, 9QOE, 9QNY, 9QNX, 9QP9, 9QOC, 9QOD, 9QOI, 9QPB, 9QOK, 9QPL, 9QOM, and 9QOS.

## References

[1] Aminov, R. History of antimicrobial drug discovery: major classes and health impact. *Biochem. Pharm.* **133**, 4–19 (2017). 27720719 10.1016/j.bcp.2016.10.001

[2] Antimicrobial Resistance Collaborators Global burden of bacterial antimicrobial resistance in 2019: a systematic analysis. *Lancet* **399**, 629–655 (2022). 35065702 10.1016/S0140-6736(21)02724-0 PMC8841637

[3] Naghavi, M. et al. Global burden of bacterial antimicrobial resistance 1990–2021: a systematic analysis with forecasts to 2050. *Lancet* **404**, 1199–1226 (2024). 39299261 10.1016/S0140-6736(24)01867-1 PMC11718157

[4] Kang, S.-J., Kim, D.-H. & Lee, B.-J. Metallo-β-lactamase inhibitors: a continuing challenge for combating antibiotic resistance. *Biophys. Chem.* **309**, 107228 (2024). 38552402 10.1016/j.bpc.2024.107228

[5] Vakulenko, S. B. & Mobashery, S. Versatility of aminoglycosides and prospects for their future. *Clin. Microbiol. Rev.* **16**, 430–450 (2003). 12857776 10.1128/CMR.16.3.430-450.2003 PMC164221

[6] Krause, K. M., Serio, A. W., Kane, T. R. & Connolly, L. E. Aminoglycosides: an overview. *Cold Spring Harb. Perspect. Med* **6**, a027029 (2016). 27252397 10.1101/cshperspect.a027029 PMC4888811

[7] Böttger, E. C. & Crich, D. Aminoglycosides: time for the resurrection of a neglected class of antibacterials? *ACS Infect. Dis.* **6**, 168–172 (2020). 31855407 10.1021/acsinfecdis.9b00441 PMC7024022

[8] Ramirez, M. S. & Tolmasky, M. E. Aminoglycoside modifying enzymes. *Drug Resist. Updat.* **13**, 151–171 (2010). 20833577 10.1016/j.drup.2010.08.003 PMC2992599

[9] Wright, G. D. Aminoglycoside-modifying enzymes. *Curr. Opin. Microbiol.* **2**, 499–503 (1999). 10508725 10.1016/s1369-5274(99)00007-7

[10] Wright, G. D. & Thompson, P. R. Aminoglycoside phosphotransferases: proteins, structure, and mechanism. *Front. Biosci.* **4**, D9–D21 (1999). 9872733 10.2741/wright

[11] Scheeff, E. D. & Bourne, P. E. Structural evolution of the protein kinase-like superfamily. *PLoS Comput. Biol.* **1**, e49 (2005). 16244704 10.1371/journal.pcbi.0010049 PMC1261164

[12] Daigle, D. M., McKay, G. A. & Wright, G. D. Inhibition of aminoglycoside antibiotic resistance enzymes by protein kinase inhibitors. *J. Biol. Chem.* **272**, 24755–24758 (1997). 9312069 10.1074/jbc.272.40.24755

[13] Burk, D. L. & Berghuis, A. M. Protein kinase inhibitors and antibiotic resistance. *Pharmacol. Therapeutics* **93**, 283–292 (2002). 10.1016/s0163-7258(02)00197-3 12191620

[14] Fong, D. H., Xiong, B., Hwang, J. & Berghuis, A. M. Crystal structures of two aminoglycoside kinases bound with a eukaryotic protein kinase inhibitor. *PLoS ONE* **6**, e19589 (2011). 21573013 10.1371/journal.pone.0019589 PMC3090406

[15] Shi, K., Houston, D. R. & Berghuis, A. M. Crystal structures of antibiotic-bound complexes of aminoglycoside 2”-phosphotransferase IVa highlight the diversity in substrate binding modes among aminoglycoside kinases. *Biochemistry* **50**, 6237–6244 (2011). 21678960 10.1021/bi200747f

[16] Toth, M., Frase, H., Antunes, N. T., Smith, C. A. & Vakulenko, S. B. Crystal structure and kinetic mechanism of aminoglycoside phosphotransferase-2”-IVa. *Protein Sci.* **19**, 1565–1576 (2010). 20556826 10.1002/pro.437 PMC2923509

[17] Shakya, T. & Wright, G. D. Nucleotide selectivity of antibiotic kinases. *Antimicrob. Agents Chemother.* **54**, 1909–1913 (2010). 20231391 10.1128/AAC.01570-09 PMC2863675

[18] Giordanetto, F., Jin, C., Willmore, L., Feher, M. & Shaw, D. E. Fragment hits: what do they look like and how do they bind? *J. Med. Chem.* **62**, 3381–3394 (2019). 30875465 10.1021/acs.jmedchem.8b01855 PMC6466478

[19] Marshall, C. M., Federice, J. G., Bell, C. N., Cox, P. B. & Njardarson, J. T. An update on the nitrogen heterocycle compositions and properties of U.S. FDA-approved pharmaceuticals (2013–2023). *J. Med. Chem.* **67**, 11622–11655 (2024). 38995264 10.1021/acs.jmedchem.4c01122

[20] Osborne, J., Panova, S., Rapti, M., Urushima, T. & Jhoti, H. Fragments: where are we now? *Biochem. Soc. Trans.* **48**, 271–280 (2020). 31985743 10.1042/BST20190694

[21] Bon, M., Bilsland, A., Bower, J. & McAulay, K. Fragment-based drug discovery—the importance of high-quality molecule libraries. *Mol. Oncol.* **16**, 3761–3777 (2022). 35749608 10.1002/1878-0261.13277 PMC9627785

[22] Cherry, M. & Mitchell, T. in *Fragment-based Drug Discovery: A Practical Approach* (eds Zartler, E. R. & Shapiro, M.) (Wiley, 2008).

[23] Erlanson, D. A., Fesik, S. W., Hubbard, R. E., Jahnke, W. & Jhoti, H. Twenty years on: the impact of fragments on drug discovery. *Nat. Rev. Drug Discov.* **15**, 605–619 (2016). 27417849 10.1038/nrd.2016.109

[24] WHO Bacterial Priority Pathogens List 2024*: Bacterial Pathogens of Public Health Importance, to Guide Research, Development, and Strategies to Prevent and Control Antimicrobial Resistance* (World Health Organization, 2024).

[25] Buffa, V. et al. Targeting bacterial kinases as a strategy to counteract antibiotic resistance. *Commun. Chem*. 10.1038/s42004-025-01794-7 (2025). 10.1038/s42004-025-01794-7 PMC12678819 41345223

[26] Lund, D. et al. Extensive screening reveals previously undiscovered aminoglycoside resistance genes in human pathogens. *Commun. Biol.* **6**, 812 (2023). 37537271 10.1038/s42003-023-05174-6 PMC10400643

[27] Toth, M., Chow, J. W., Mobashery, S. & Vakulenko, S. B. Source of phosphate in the enzymic reaction as a point of distinction among aminoglycoside 2”-phosphotransferases. *J. Biol. Chem.* **284**, 6690–6696 (2009). 19158087 10.1074/jbc.M808148200 PMC2652286

[28] Siregar, J. J., Lerner, S. A. & Mobasheryi, S. Purification and characterization of aminoglycoside 3’-phosphotransferase type Ila and kinetic comparison with a new mutant enzyme. *Antimicrob. Agents Chemother.* **38**, 641–647 (1994). 8031025 10.1128/aac.38.4.641 PMC284518

[29] Kowalewski, J. et al. Structural and molecular basis of resistance to aminoglycoside antibiotics by APH(3’)-IIb from *Pseudomonas aeruginosa*. 10.1101/2025.03.30.646141 (2025).

[30] Fong, D. H. & Berghuis, A. M. Crystallization and preliminary crystallographic analysis of 3’-aminoglycoside kinase type IIIa complexed with a eukaryotic protein kinase inhibitor, CKI-7. *Acta Crystallogr. D: Biol. Crystallogr.* **60**, 1897–1899 (2004). 15388945 10.1107/S0907444904019390

[31] Stogios, P. J. et al. Structure-guided optimization of protein kinase inhibitors reverses aminoglycoside antibiotic resistance. *Biochem. J.* **454**, 191–200 (2013). 23758273 10.1042/BJ20130317 PMC3743924

[32] Boehr, D. D., Lane, W. S. & Wright, G. D. Active site labeling of the gentamicin resistance enzyme AAC(6’)-APH(2”) by the lipid kinase inhibitor wortmannin. *Chem. Biol.* **8**, 791–800 (2001). 11514228 10.1016/s1074-5521(01)00051-5

[33] Kohl, A. et al. Allosteric inhibition of aminoglycoside phosphotransferase by a designed ankyrin repeat protein. *Structure* **13**, 1131–1141 (2005). 16084385 10.1016/j.str.2005.04.020

[34] Leban, N., Kaplan, E., Chaloin, L., Godreuil, S. & Lionne, C. Kinetic characterization and molecular docking of novel allosteric inhibitors of aminoglycoside phosphotransferases. *Biochim. Biophys. Acta Gen. Subj.* **1861**, 3464–3473 (2017). 27640112 10.1016/j.bbagen.2016.09.012

[35] Kaplan, E. et al. APH inhibitors that reverse aminoglycoside resistance in *Enterococcus casseliflavus*. *ChemMedChem* **20**, e202400842 (2025) 10.1002/cmdc.202400842 PMC12005471 39801466

[36] Reys, V., Kowalewski, J., Gelin, M. & Lionne, C. w*TSA-CRAFT*: an open-access web server for rapid analysis of thermal shift assay experiments. *Bioinform Adv.* **3**, vbad136 (2023). 37822724 10.1093/bioadv/vbad136 PMC10562953

[37] Kaplan, E. et al. Aminoglycoside binding and catalysis specificity of aminoglycoside 2”-phosphotransferase IVa: A thermodynamic, structural and kinetic study. *Biochim. Biophys. Acta (BBA) - Gen. Subj.* **1860**, 802–813 (2016). 10.1016/j.bbagen.2016.01.016 PMC4769084 26802312

[38] Emsley, P., Lohkamp, B., Scott, W. G. & Cowtan, K. Features and development of Coot. *Acta Crystallogr. D: Biol. Crystallogr.* **66**, 486–501 (2010). 20383002 10.1107/S0907444910007493 PMC2852313

[39] Afonine, P. V. et al. Towards automated crystallographic structure refinement with phenix.refine. *Acta Crystallogr D: Biol. Crystallogr.* **68**, 352–367 (2012). 22505256 10.1107/S0907444912001308 PMC3322595

