# Supplementary material for: Fragment-based drug design of a bacterial kinase inhibitor capable of increasing the antibiotic sensitivity of clinical isolates
Source: Commun Chem. 2025 Nov 27;8:417. doi: 10.1038/s42004-025-01795-6 (PMC12749166; doi:10.1038/s42004-025-01795-6)
Supplement: Supplementary file 1 — Description of Additional Supplementary Files [file 42004_2025_1795_MOESM1_ESM.pdf]

Description of Additional Supplementary Files

File name- Supplemental Data

File description – Source data underlying the graphs and charts presented in the figures have been uploaded as Supplementary Data in one Excel file.
