# Supplementary material for: Fragment-based drug design of a bacterial kinase inhibitor capable of increasing the antibiotic sensitivity of clinical isolates
Source: Commun Chem. 2025 Nov 27;8:417. doi: 10.1038/s42004-025-01795-6 (PMC12749166; doi:10.1038/s42004-025-01795-6)
Supplement: Supplementary file 2 — Supplementary Information [file 42004_2025_1795_MOESM2_ESM.pdf]

Supplementary information

Fragment-based drug design of a bacterial kinase inhibitor capable of increasing the antibiotic sensitivity of clinical isolates

Julien Kowalewski[1], Robin Deutscher[2], Marion Richardoz[3], Mathilde Tomaszczyk[1], Muriel Gelin[1], Gilles Labesse[1], Felix Hausch[2,4], Gerard D. Wright[5], Catherine Dunyach-Remy[3], Jean-François Guichou[1], Corinne Lionne[1,*]

[1-5] See full list of affiliations in the article.
* Corresponding author: corinne.lionne@cnrs.fr

Supplementary Figure 1. Screening of the effect of 389 fragments on the thermostability and *in vitro* enzymatic activity of APH(2'')-IVa.

Supplementary Figure 2. Crystal structures and corresponding omit maps contoured at a sigma level of ± 1 of APH(2'')-IVa in complex with **F136** and **F355**.

Supplementary Figure 3. Characterization of the effect of **83** on the growth of *P. aeruginosa* C0307 and *S. aureus* C0032 after 18 h incubation.

Supplementary Figure 4. Characterization of the effect of **83** on the growth kinetics of different *P. aeruginosa* clinical isolates.

Supplementary Tables 1-5. Data collection and refinement statistics (molecular replacement).

Supplementary Table 6. Characteristics of clinical isolates used in this work.

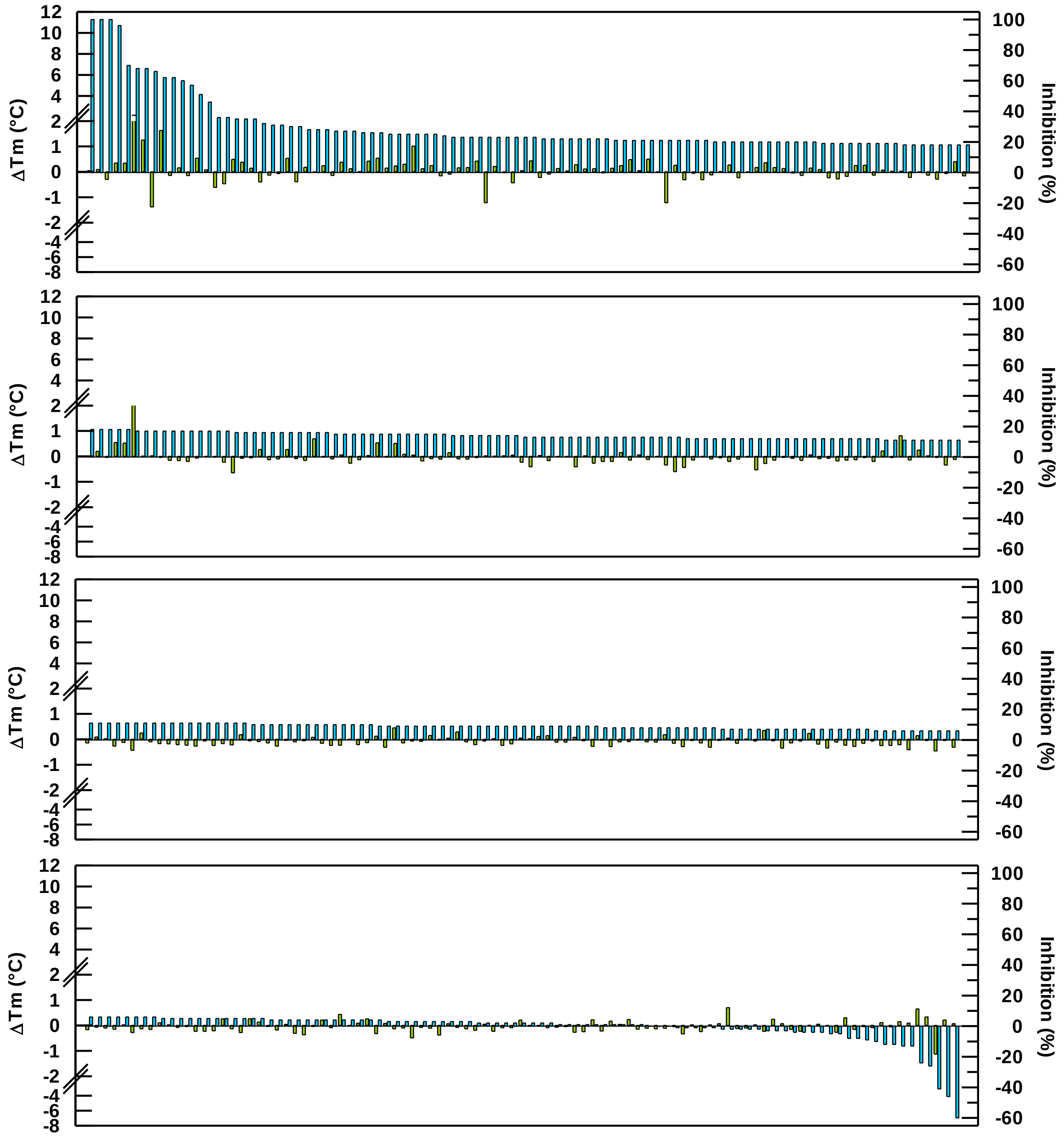

Supplementary Figure 1. **Screening of the effect of 389 fragments on the thermostability and *in vitro* enzymatic activity of APH(2'')-IVa.** For TSA assays (green bars), final concentrations were 5 µM APH(2'')-IVa, 500 µM fragments and 5× Sypro Orange. For activity measurements (blue bars), final concentrations were 0.1 µM APH(2'')-IVa, 350 µM MgATP, 100 µM kanamycin A, 500 µM fragments, 2 mM PEP, 140 µM NADH, and 1× LDH/PK. Fragments are ranked according to the inhibition they induce, from the largest (top) to the smallest (bottom). Some values may be distorted due to the absorbance or fluorescence of some compounds.

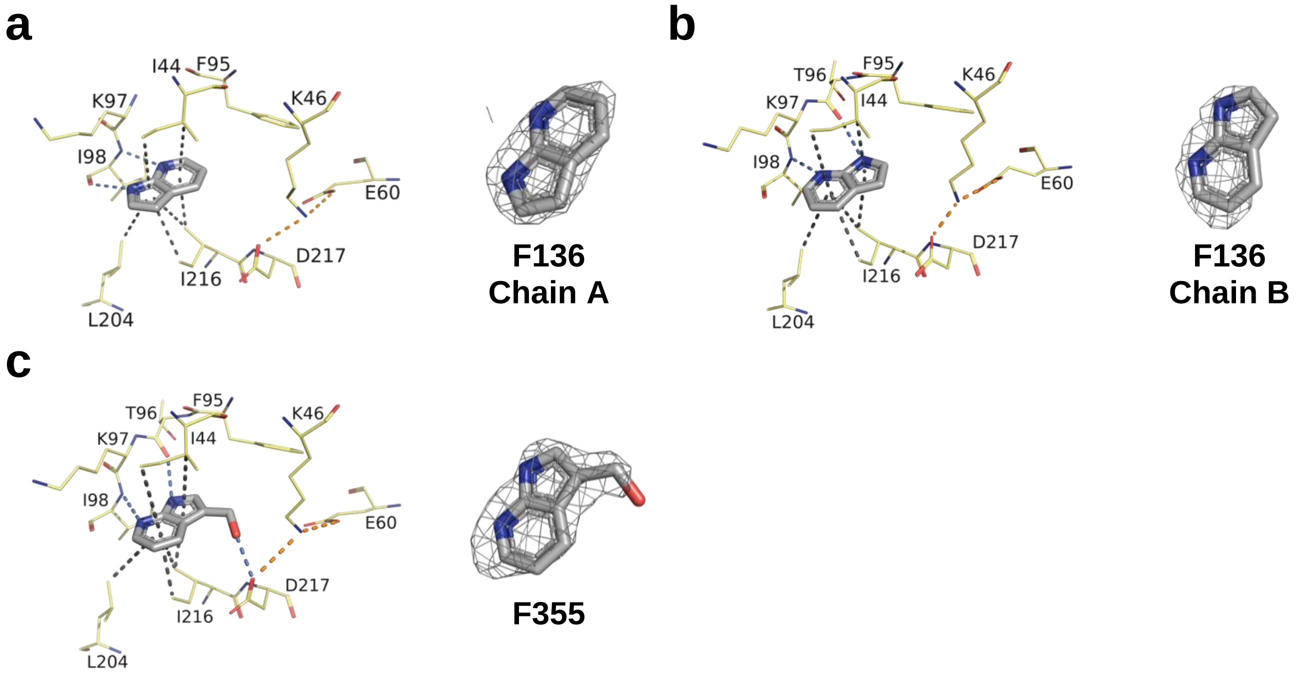

Supplementary Figure 2. **Crystal structures and corresponding omit maps contoured at a sigma level of ± 1 of APH(2'')-IVa in complex with F136 and F355.** (**a**, **b**) Structure of the complex of APH(2'')-IVa with **F136** determined at 2.30 Å with best predicted, but probably alternating, orientations in the A and B chains, respectively (PDB 9QPD). (**c**) Structure of the complex of APH(2'')-IVa with **F355** at 2.30 Å (PDB 9QN6). Inhibitors are represented in grey sticks and residues involved in interactions are shown as yellow lines. Interactions are shown as dashed lines: van der Waals interactions in grey, hydrogen bonds in blue and ionic bonds in orange.

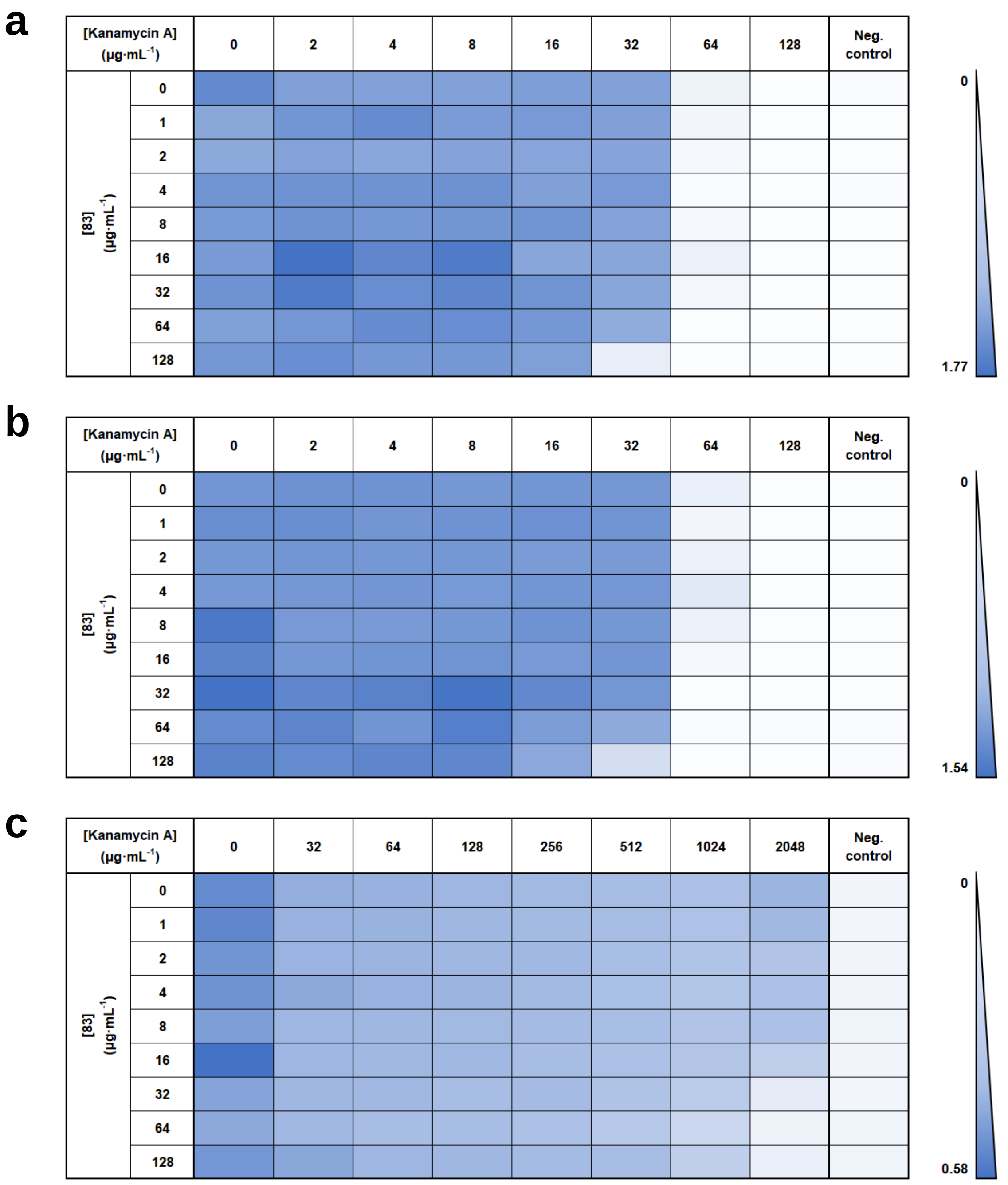

Supplementary Figure 3. Characterization of the effect of 83 on the growth of *P. aeruginosa* C0307 and *S. aureus* C0032 after 18 h incubation. Measurement of the OD_{600nm} after 18 h at 37°C of *P. aeruginosa* C0307 (**a**) in the absence or (**b**) in the presence of 0.05 µg.mL^{-1} of imipenem or for *S. aureus* C0032 (**c**) in the presence of 0.125 µg.mL^{-1} of imipenem. Cells of the table are stained with a linear color gradient according to OD_{600nm}, from white at 0 to dark blue at the maximum value in the plate, indicated next to the color scale. The first column shows the lack of effect of **83** in the absence of kanamycin A. The last column represents the negative control (medium with **83** but without bacteria).

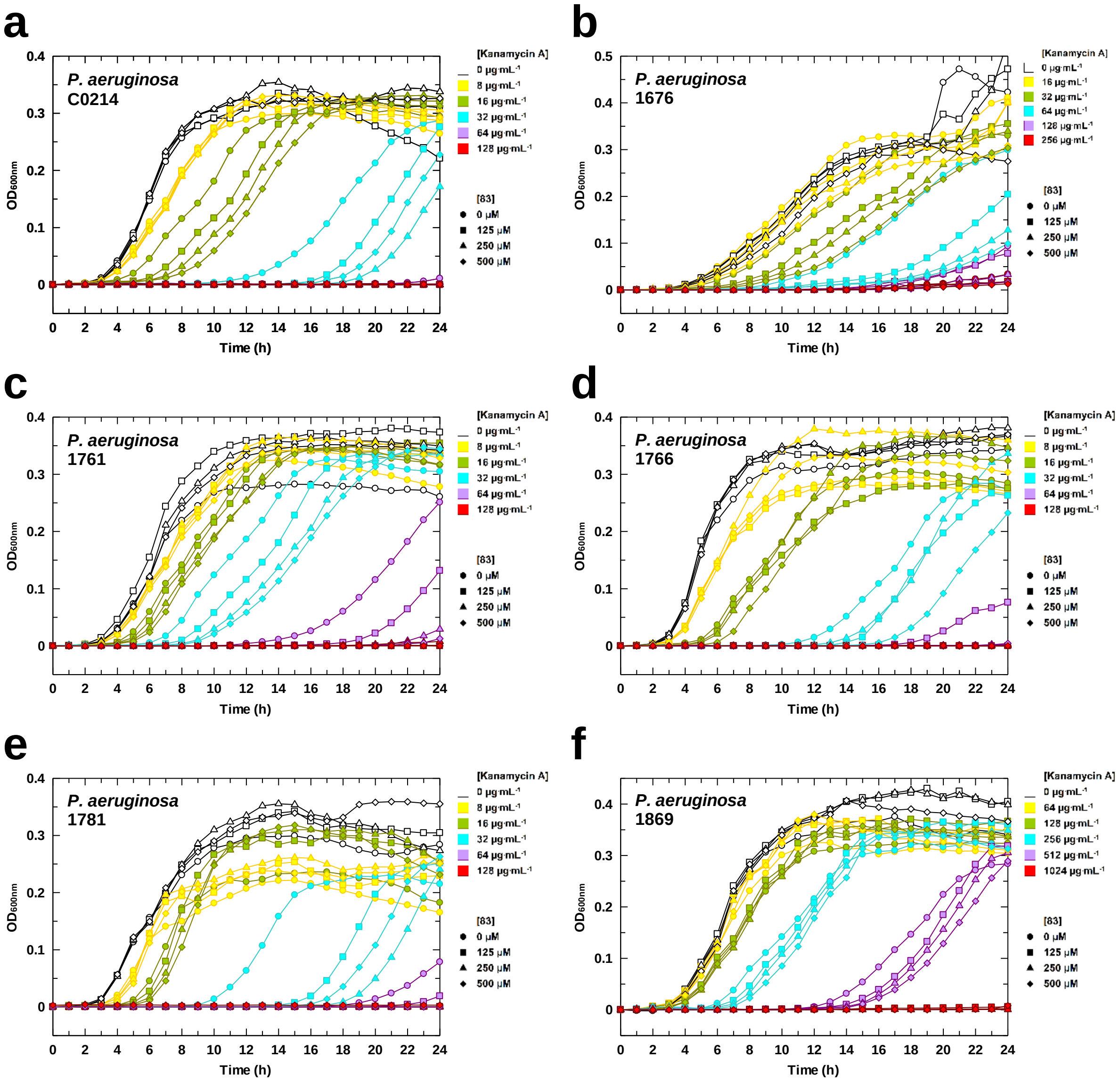

Supplementary Figure 4. Characterization of the effect of 83 on the growth kinetics of different *P. aeruginosa* clinical isolates. Measurement of the 24 h growth kinetics of *P. aeruginosa* (**a**) C0214, (**b**) 1676, (**c**) 1761, (**d**) 1766, (**e**) 1781, and (**f**) 1869 in the presence of increasing concentrations of kanamycin A (different colors) and different concentrations of **83** (different symbols). The characteristics of these strains are shown in the Supp. Table 6. The symbols circles, squares, triangles and diamonds correspond respectively to 0, 125, 250 and 500 µM of **83**. The colors black, yellow, green, cyan, mauve and red correspond respectively to 0, 8, 16, 32, 64 and 128 µg.mL^{-1} of Kanamycin A in (**a**, **c**, **d** and **e**), 0, 16, 32, 64, 128 and 256 µg.mL^{-1} of Kanamycin A in (**b**) and 0, 64, 128, 256, 512 and 1024 µg.mL^{-1} of Kanamycin A in (**f**).

Supplementary Table 1. Data collection and refinement statistics (molecular replacement).

(PDB accession code)	APH(2'')-IVa-F69 (9QNQ)	APH(2'')-IVa-F136 (9QPD)	APH(2'')-IVa-F245 (9QNS)	APH(2'')-IVa-F274 (9QMR)
**Data collection**				
Space group	$P2_1$	$P2_1$	$P2_1$	$P2_1$
Cell dimensions				
a, *b*, *c* (Å)	75.78 65.12 76.90	79.11 64.55 72.33	75.72 64.67 76.34	75.28 65.43 77.47
α, β, γ (°)	90 91.7 90	90 91.0 90	90 92.0 90	90 91.7 90
Resolution (Å)	49.69 -2.38 (2.46-2.38)*	52.91 -2.30 (2.38-2.30)*	49.34-2.00 (2.07-2.00)*	41.35 -1.78 (1.84-1.78)*
R_{merge}	0.099 (0.708)	0.047 (0.420)	0.098 (0.624)	0.031 (0.570)
R_{means}	0.111 (0.792)	0.066 (0.595)	0.118 (0.760)	0.038 (0.693)
R_{pim}	0.049 (0.346)	0.047 (0.420)	0.065 (0.429)	0.021 (0.388)
$I / \sigma I$	10.12 (2.42)	11.13 (1.82)	14.02 (1.89)	15.88 (1.80)
Completeness (%)	99.62 (99.40)	96.04 (97.62)	98.78 (98.79)	98.84 (99.65)
Redundancy	4.8 (5.0)	1.8 (1.8)	3.0 (2.9)	3.0 (3.0)
$CC_{1/2}$	0.995 (0.808)	0.962 (0.629)	0.954 (0.715)	0.999 (0.738)
CC^*	0.999 (0.946)	0.990 (0.879)	0.988 (0.913)	1.000 (0.922)
Wilson B-factor	40.66	49.52	41.43	37.19
**Refinement**				
Resolution (Å)	2.38	2.30	2.00	1.78
No. reflections	145397 (14894)	55457 (5681)	147675 (14117)	214998 (21730)
Unique reflections	30207 (2999)	31349 (3155)	49652 (4883)	71454 (7176)
R_{work} / R_{free}	22.30/29.61	19.78/24.65	21.62/25.60	19.60/22.57
No. atoms				
Protein	4927	4795	4723	4944
Ligand/ion	28	22	104	62
Water	134	51	116	199
B-factors				
Protein	48.84	60.69	52.96	46.63
Ligand/ion	43.61	54.87	53.07	56.18
Water	41.06	55.49	46.66	46.18
R.m.s. deviations				
Bond lengths (Å)	0.009	0.009	0.009	0.008
Bond angles (°)	0.98	1.01	1.78	0.98
Number of TLS	8	9	9	5

*Values in parentheses are for the highest-resolution shell.

Supplementary Table 2. Data collection and refinement statistics (molecular replacement).

(PDB accession code)	APH(2'')-IVa-F355 (9QN6)	APH(2'')-IVa-F382 (9QNN)	APH(2'')-IVa-1 (9QNW)	APH(2'')-IVa-2 (9QOK)
**Data collection**				
Space group	$P2_1$	$P2_1$	$P2_1$	$P2_1$
Cell dimensions				
a, *b*, *c* (Å)	76.31 65.39 77.42	75.38 65.09 76.94	73.82 65.77 77.62	77.39 65.26 74.27
α, β, γ (°)	90 91.4 90	90 90.7 90	90 91.2 90	90 91.2 90
Resolution (Å)	49.95-2.30 (2.38-2.30)*	37.69 -2.65 (2.75-2.65)*	34.64-2.47 (2.56-2.47)*	41.68 -2.02 (2.09-2.02)*
R_{merge}	0.054 (0.382)	0.074 (0.937)	0.044 (0.492)	0.063 (0.896)
R_{means}	0.067 (0.466)	0.089 (1.124)	0.054 (0.599)	0.078 (1.110)
R_{pim}	0.038 (0.263)	0.049 (0.611)	0.030 (0.336)	0.045 (0.644)
$I / \sigma I$	13.44 (2.81)	12.06 (1.30)	14.44 (2.49)	8.85 (1.23)
Completeness (%)	94.27 (98.32)	92.67 (92.58)	85.67 (89.83)	96.83 (98.02)
Redundancy	2.9 (3.0)	2.9 (3.0)	2.7 (2.7)	3.1 (3.1)
$CC_{1/2}$	0.995 (0.914)	0.998 (0.689)	0.999 (0.866)	0.995 (0.658)
$CC^{*0.996\ (0.859)}$	0.999 (0.977)	1.000 (0.903)	1.000 (0.964)	0.999 (0.891)
Wilson B-factor	37.25	64.07	53.18	44.35
**Refinement**				
Resolution (Å)	2.30	2.65	2.47	2.02
No. reflections	98005 (10086)	58951 (6109)	61543 (6311)	131790 (12940)
Unique reflections	33269 (3373)	20278 (1995)	22796 (2316)	47236 (4745)
R_{work} / R_{free}	22.28/27.80	20.56/28.21	20.13/26.87	21.73/25.80
No. atoms				
Protein	4922	4922	4914	4789
Ligand/ion	26	19	16	55
Water	99	23	24	41
B-factors				
Protein	48.73	70.75	66.64	55.62
Ligand/ion	39.91	55.61	55.16	52.76
Water	40.83	51.38	48.05	47.50
R.m.s. deviations				
Bond lengths (Å)	0.008	0.010	0.009	0.008
Bond angles (°)	0.93	1.10	1.08	0.94
Number of TLS	15	8	15	9

*Values in parentheses are for the highest-resolution shell.

Supplementary Table 3. Data collection and refinement statistics (molecular replacement).

(PDB accession code)	APH(2'')-IVa-3 (9QPB)	APH(2'')-IVa-4 (9QOL)	APH(2'')-IVa-5 (9QOM)	APH(2'')-IVa-11 (9QNY)
**Data collection**				
Space group	$P2_1$	$P2_1$	$P2_1$	$P2_1$
Cell dimensions				
a, *b*, *c* (Å)	61.62 63.57 87.00	77.92 65.63 75.82	76.00 65.05 78.35	75.87 65.43 77.07
α, β, γ (°)	90 90.2 90	90 91.7 90	90 91.6 90	90 91.6 90
Resolution (Å)	39.49-1.86 (1.92-1.86)*	50.19-2.13 (2.20-2.13)*	75.97-2.13 (2.20-2.13)*	49.46 -2.41 (2.50-2.41)*
R_{merge}	0.079 (1.006)	0.060 (1.069)	0.085 (1.283)	0.065 (0.549)
R_{means}	0.094 (1.202)	0.074 (1.302)	0.105 (1.568)	0.080 (0.666)
R_{pim}	0.050 (0.651)	0.042 (0.733)	0.061 (0.890)	0.045 (0.371)
$I / \sigma I$	9.12 (1.25)	9.61 (1.24)	10.08 (0.86)	11.12 (1.97)
Completeness (%)	97.83 (96.37)	93.41 (89.48)	92.88 (94.39)	95.31 (98.89)
Redundancy	3.4 (3.3)	2.8 (2.8)	2.9 (2.9)	3.0 (3.1)
$CC_{1/2}$	0.996 (0.442)	0.997 (0.427)	0.976 (0.460)	0.997 (0.848)
$CC^{*0.996\ (0.859)}$	0.999 (0.783)	0.999 (0.774)	0.994 (0.794)	0.999 (0.958)
Wilson B-factor	31.05	41.74	38.23	50.78
**Refinement**				
Resolution (Å)	1.86	2.13	2.13	2.41
No. reflections	186407 (17946)	113283 (11915)	117646 (12229)	82823 (8833)
Unique reflections	55495 (5471)	41169 (9811)	41241 (4035)	27885 (2866)
R_{work} / R_{free}	21.10/25.21	21.63/25.21	22.45/27.28	21.38/28.60
No. atoms				
Protein	4689	4860	4884	4943
Ligand/ion	115	20	28	40
Water	148	43	77	52
B-factors				
Protein	36.39	51.97	50.63	61.46
Ligand/ion	41.33	43.02	56.93	57.17
Water	34.55	41.86	40.67	50.31
R.m.s. deviations				
Bond lengths (Å)	0.009	0.008	0.008	0.009
Bond angles (°)	1.00	0.96	1.00	1.04
Number of TLS	15	8	12	8

*Values in parentheses are for the highest-resolution shell.

Supplementary Table 4. Data collection and refinement statistics (molecular replacement).

(PDB accession code)	APH(2'')-IVa-36 (9QOE)	APH(2'')-IVa-36-3 (9QNX)	APH(2'')-IVa-36-5 (9QP9)	APH(2'')-IVa-36-9 (9QPL)
**Data collection**				
Space group	$P2_1$	$P2_1$	$P2_1$	$P2_1$
Cell dimensions				
a, *b*, *c* (Å)	76.17 65.58 78.06	74.99 65.24 77.16	75.69 64.74 76.39	75.84 65.09 77.16
α, β, γ (°)	90 92.1 90	90 91.6 90	90 92.0 90	90 91.9 90
Resolution (Å)	41.45-2.30 (2.38-2.30)*	49.82-2.11 (2.18-2.11)*	75.65-2.30 (2.38-2.30)*	75.80 -2.04 (2.11-2.04)*
R_{merge}	0.060 (0.591)	0.164 (0.784)	0.109 (0.260)	0.084 (0.714)
R_{means}	0.067 (0.668)	0.199 (0.951)	0.132 (0.312)	0.103 (0.903)
R_{pim}	0.030 (0.302)	0.111 (0.531)	0.072 (0.170)	0.059 (0.545)
$I / \sigma I$	13.87 (2.34)	4.14 (0.93)	23.91 (2.41)	6.62 (1.11)
Completeness (%)	98.52 (99.45)	95.93 (93.05)	99.31 (99.73)	99.16 (98.52)
Redundancy	4.6 (4.6)	3.1 (3.1)	3.1 (3.2)	2.8 (2.5)
$CC_{1/2}$	0.999 (0.862)	0.928 (0.490)	0.968 (0.897)	0.988 (0.557)
$CC^{*0.996\ (0.859)}$	1.000 (0.962)	0.981 (0.811)	0.992 (0.972)	0.997 (0.846)
Wilson B-factor	47.69	35.10	37.00	38.26
**Refinement**				
Resolution (Å)	2.30	2.11	2.30	2.04
No. reflections	156333 (15678)	131084 (12288)	101203 (10610)	133663 (11876)
Unique reflections	33959 (3435)	42173 (3978)	32862 (3299)	47660 (4674)
R_{work} / R_{free}	20.58/25.37	24.93/29.15	19.49/25.19	20.61/23.94
No. atoms				
Protein	4946	4900	4826	4864
Ligand/ion	32	52	62	74
Water	84	146	173	166
B-factors				
Protein	63.43	48.69	47.54	45.82
Ligand/ion	58.37	48.41	47.22	45.88
Water	55.17	44.35	42.23	42.91
R.m.s. deviations				
Bond lengths (Å)	0.008	0.008	0.007	0.008
Bond angles (°)	0.95	0.99	0.90	0.89
Number of TLS	6	7	6	8

*Values in parentheses are for the highest-resolution shell.

Supplementary Table 5. Data collection and refinement statistics (molecular replacement).

(PDB accession code)	APH(2'')-IVa-68 (9QOI)	APH(2'')-IVa-83 (9QOD)	APH(2'')-IVa-85 (9QOC)	APH(3')-IIb-83 (9QOS)
**Data collection**				
Space group	$P2_1$	$P2_1$	$P2_1$	$C222_1$
Cell dimensions				
a, *b*, *c* (Å)	75.77 64.56 76.98	75.82 65.27 77.62	75.25 64.48 77.31	83.50 109.66 124.41
α, β, γ (°)	90 91.6 90	90 91.5 90	90 91.6 90	90 90 90
Resolution (Å)	41.08 -1.92 (1.99-1.92)*	49.95 -2.29 (2.37-2.29)*	48.96-1.99 (2.06-1.99)*	32.34 -1.97 (2.04-1.97)*
R_{merge}	0.049 (0.684)	0.052 (0.255)	0.031 (0.381)	0.088 (0.725)
R_{means}	0.060 (0.842)	0.063 (0.307)	0.037 (0.459)	0.124 (1.026)
R_{pim}	0.034 (0.484)	0.034 (0.169)	0.020 (0.252)	0.088 (0.725)
$I / \sigma I$	12.34 (1.57)	14.55 (4.18)	19.10 (3.01)	7.39 (1.08)
Completeness (%)	99.14 (98.94)	99.34 (98.74)	98.33 (97.46)	97.36 (98.43)
Redundancy	3.0 (2.8)	3.1 (3.2)	3.2 (3.1)	1.9 (1.9)
$CC_{1/2}$	0.999 (0.700)	0.998 (0.954)	0.999 (0.914)	0.993 (0.444)
$CC^{*0.996\ (0.859)}$	0.999 (0.908)	0.999 (0.988)	1.000 (0.977)	0.998 (0.784)
Wilson B-factor	37.37	35.46	37.93	26.72
**Refinement**				
Resolution (Å)	1.92	2.29	1.99	1.97
No. reflections	167236 (15544)	107077 (10660)	158074 (15447)	75586 (7607)
Unique reflections	56481 (5598)	34108 (3374)	50014 (4922)	39616 (3960)
R_{work} / R_{free}	19.48/23.06	18.68/23.74	19.14/23.23	22.28/25.28
No. atoms				
Protein	4845	4908	4938	3997
Ligand/ion	52	38	62	30
Water	226	167	207	260
B-factors				
Protein	47.72	48.62	48.13	31.86
Ligand/ion	50.74	43.79	48.03	26.67
Water	46.03	41.51	46.45	31.06
R.m.s. deviations				
Bond lengths (Å)	0.009	0.008	0.009	0.08
Bond angles (°)	1.01	0.90	0.98	0.98
Number of TLS	11	7	10	8

*Values in parentheses are for the highest-resolution shell.

Supplementary Table 6. Characteristics of clinical isolates used in this work. The origin of the bacteria and the AME genes expressed are given. MICs of antibiotics are indicated in μg.mL^{-1}.

**Bacteria**	**Reference**	**Origin**[a]	**AME genes**	**MIC aminoglycosides (μg.mL^{-1})**				**MIC carbapenems (μg.mL^{-1})**			
				**Amikacin**	**Gentamicin**	**Kanamycin A**	**Streptomycin**	**Doripenem**	**Ertapenem**	**Imipenem**	**Meropenem**
P. aeruginosa	C0214	HGH	*aph(3')-IIb*	8-32	2	256-512	32	0.5-1	> 2	> 2	0.5-1
	C0307	HGH	*aph(3')-IIb*	4-8	2	64-128	64	0.25	8-16	2	0.063-0.125
	1676	NH	*aph(3')-IIb*	nd	nd	128	nd	nd	nd	nd	nd
	1761	NH	*aph(3')-IIb*	nd	nd	128	nd	nd	nd	nd	nd
	1766	NH	*aph(3')-IIb*	nd	nd	128	nd	nd	nd	nd	nd
	1781	NH	*aph(3')-IIb*	nd	nd	512	nd	nd	nd	nd	nd
	1869	NH	*aph(3')-IIb*<br>*aac(6')-Ib3*	nd	nd	1024	nd	nd	nd	nd	nd
S. aureus	C0032	HGH	*aph(3')-IIIa*<br>*ant(6)*	16-32	< 1	4096	2-4	> 4	4	2-1	> 4

[a] HGH = Hamilton General Hospital, ON, Canada; NH = Nîmes Hospital, Nîmes, France. nd = not determined.
